# Soluble epoxide hydrolase maintains steady-state lipid turnover linked with autocrine signaling in peritoneal macrophages

**DOI:** 10.1016/j.isci.2023.107465

**Published:** 2023-07-27

**Authors:** Feng Liu, Xueying Diao, Haolun Cong, Eriko Suzuki, Keiji Hasumi, Hiroshi Takeshima

**Affiliations:** 1 Graduate School of Pharmaceutical Sciences, Kyoto University, Kyoto 606-8501, Japan; 2 Department of Applied Biological Science, Tokyo University of Agriculture and Technology, Tokyo 183-8509, Japan; 3 Division of Research and Development, TMS Co., Ltd, Tokyo 183-0023, Japan

**Keywords:** Biological sciences, Biochemistry, Immunology, Cell biology

## Abstract

Soluble epoxide hydrolase is a widely distributed bifunctional enzyme that contains N-terminal phosphatase (N-phos) and C-terminal epoxide hydrolase (C-EH) domains. C-EH hydrolyzes anti-inflammatory epoxy-fatty acids to corresponding diols and contributes to various inflammatory conditions. However, N-phos has been poorly examined. In peritoneal macrophages, the N-phos inhibitor amino-hydroxybenzoic acid (AHBA) seemed to primarily interrupt the dephosphorylation of lysophosphatidates and broadly attenuated inflammation-related functions. AHBA activated intrinsic lysophosphatidate and thromboxane A2 receptors by altering lipid-metabolite distribution; downstream the signaling, phospholipase C was facilitated to dampen intracellular Ca^2+^ stores and AKT kinase (protein kinase B) was activated to presumably inhibit inflammatory gene expression. Our data suggest that N-phos maintains steady-state phospholipid turnover connecting autocrine signaling and is a prospective target for controlling inflammatory responses in macrophages.

## Highlights

• AHBA-mediated N-phos inhibition altered phospholipid metabolism in macrophages
• AHBA autocrine-activated lysophosphatidate and thromboxane A2 receptors in macrophages
• AHBA-induced autocrine signaling inhibited inflammation-related functions in macrophages
• AHBA attenuated M1 polarization in cultured bone marrow-derived macrophages

## Graphical abstract

**Figure:**
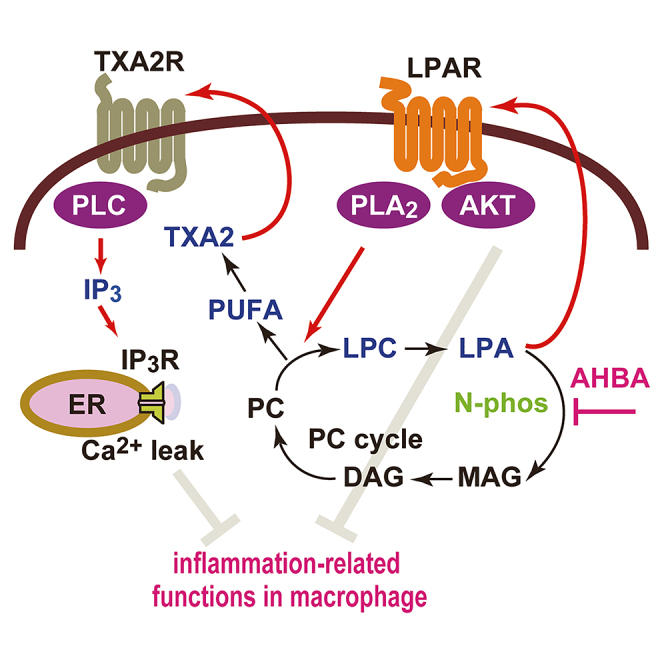

## Introduction

Polyunsaturated fatty acids (PUFAs), such as arachidonic and eicosapentaenoic acids, are converted into lipid mediators by cyclooxygenase (COX), lipoxygenase (LOX), and cytochrome P450.^1^ COX and LOX produce prostanoid and eicosanoid precursors, while P450 monooxygenase generates anti-inflammatory epoxy-fatty acids, which are further metabolized into pro-inflammatory acyl diols by soluble epoxide hydrolase (sEH).^2^ Therefore, sEH inhibition is thought to exert anti-inflammatory effects via the accumulation of epoxidated fatty acids such as epoxy-eicosatrienoic and epoxy-docosapentaenoic acids.^2^ Indeed, sEH inhibitors and epoxy-fatty acid analogs develop beneficial effects on various inflammation-related disease models including tissue flares, hypertension, cardiac hypertrophy, hyperglycemia, and hyperlipidemia.^2^^,^^3^

In mammals, sEH is broadly expressed in various cell types of body tissues and forms a homo-dimer in the cytoplasm and peroxisome.^4^ Importantly, sEH is a bifunctional enzyme with a molecular mass of ∼62 kDa; the lipid epoxide hydrolase activity is located in its 36-kDa C-terminal domain, while the 25-kDa N-terminal domain possesses lipid phosphatase activity.^4^ As described previously, the role of the C-terminal epoxide hydrolase (C-EH) has been extensively characterized by means of several inhibitors and mutant mice in various disease models.^2^^,^^3^ However, the N-terminal phosphatase (N-phos) has been poorly examined. In *in vitro* enzyme assays, N-phos catalyzes the dephosphorylation of several phosphorylated lipids including isoprenoid pyrophosphates, lysophosphatidic acids (LPAs), and sphingosine-1-phosphate.^5^^,^^6^^,^^7^ A recent study using the knock-in rats bearing an N-phos-defective mutation indicates that N-phos mainly converts LPAs into monoacylglycerols (MAGs) in the liver.^8^ Moreover, the N-phos-defective mutation likely confers resistance to fat mass gain during high-fat feeding and heart injury induced by ischemia-reperfusion, suggesting that N-phos takes part in pathological complications.^8^ It is therefore important to examine the patho- and physiological role of N-phos using the chemical tools that selectively modulate N-phos activity. Kihara et al. currently showed that 3-amino-4-hydroxy benzoic acid (AHBA) specifically inhibits N-phos without affecting C-EH activity.^9^ In this study, we focus on AHBA-mediated N-phos inhibition in thioglycolate-elicited peritoneal macrophages (TGPMs), which are widely used as a primary-culture model for immunological studies. Our results suggest that N-phos catalyzes a key dephosphorylation to maintain steady-state phospholipid turnover, and also that AHBA-induced N-phos inhibition stimulates autocrine signaling to attenuate inflammation-related functions in TGPMs.

## Results

### AHBA reduced store Ca^2+^ contents

**Figure 1:**
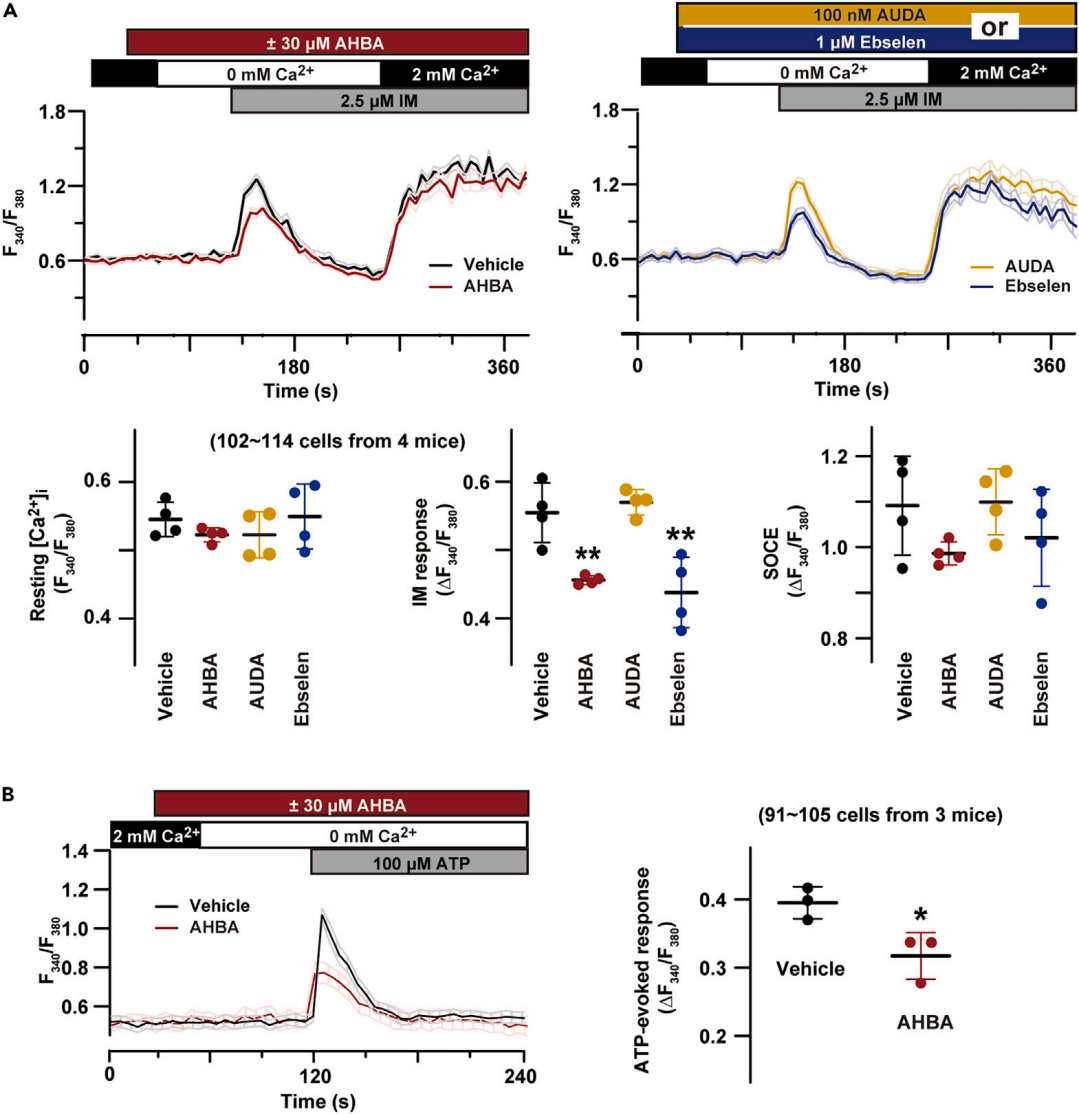
N-phos inhibition and Ca^2+^ store reduction (A) Effects of sEH inhibitors on basic Ca^2+^ handing features. The Fura-2 imaging traces show averaged time courses with shaded areas indicating standard errors (n = 10 TGPMs in each group). The Ca^2+^ ionophore ionomycin (IM) was used to trigger Ca^2+^ release and deplete intracellular stores. The inhibitors used were the N-phos-specific inhibitor AHBA, the C-EH-specific inhibitor AUDA, and the dual inhibitor ebselen. In the dot plots, resting Fura-2 ratio, ionomycin-induced response, and SOCE amplitude are statistically analyzed. The data represent means ± SEM., and the numbers of cells and mice examined are shown in parentheses. Significant differences from the vehicle-treated group are marked with asterisks (∗∗p < 0.01 in one-way ANOVA and Dunnett’s test). (B) AHBA reduces Ca^2+^ release in response to P2Y receptor activation. The imaging traces show averaged time courses with shaded areas indicating standard errors (n = 10 TGPMs in each group). In the dot plot, ATP-induced responses are statistically analyzed. A significant difference is marked with an asterisk (∗p < 0.05 in *t*-test).

We first explored the acute impact of N-phos inhibition on cellular Ca^2+^ handling in TGPMs using three chemical tools; the N-phos-selective inhibitor AHBA,^9^ the C-EH-selective inhibitor AUDA,^10^ and the dual N-phos and C-EH inhibitor ebselen.^11^ In Fura-2 ratiometric Ca^2+^ imaging, we surveyed drug-induced shifts in resting intracellular Ca^2+^ concentration ([Ca^2+^]_i_), store Ca^2+^ release triggered by the Ca^2+^ ionophore ionomycin, and store-operated Ca^2+^ entry (SOCE) in response to Ca^2+^ store depletion. AHBA (30 μM) did not affect resting [Ca^2+^]_i_ and SOCE, but significantly reduced ionomycin-induced Ca^2+^ release within a short exposure period (Figure 1A). AHBA treatment also decreased Ca^2+^ release mediated by inositol trisphosphate (IP_3_) receptors in response to ATP-induced P2Y receptor activation (Figure 1B). Ebselen reduced ionomycin-induced Ca^2+^ release as similar to AHBA, while AUDA exerted no effects on intracellular Ca^2+^ stores (Figure 1A). Therefore, independently of C-EH blockade, N-phos inhibition likely reduced the Ca^2+^ contents of IP_3_-sensitive stores in TGPMs. This consequence was also supported by imaging data using another set of inhibitors, i.e., the N-phos inhibitor oxaprozin,^12^ the C-EH inhibitor TPPU,^13^ and the dual inhibitor N-acetyl-S-farnesyl-L-cysteine^12^ (Figure S1A).

In our preliminary search, AHBA treatment also reduced Ca^2+^ stores in the Raw 264.7 macrophage cell line, A20 lymphoma cell line, primary-cultured mouse embryonic fibroblasts and hepatocytes dissociated from adult mice (Figure S1B). Therefore, acute N-phos inhibition seemed to shrink intracellular Ca^2+^ stores in various cell types. We then attempted to clarify the molecular mechanism underlying the AHBA-induced shrinkage of Ca^2+^ stores in TGPMs.

### AHBA PLC-dependently facilitated store Ca^2+^ leakage

**Figure 2:**
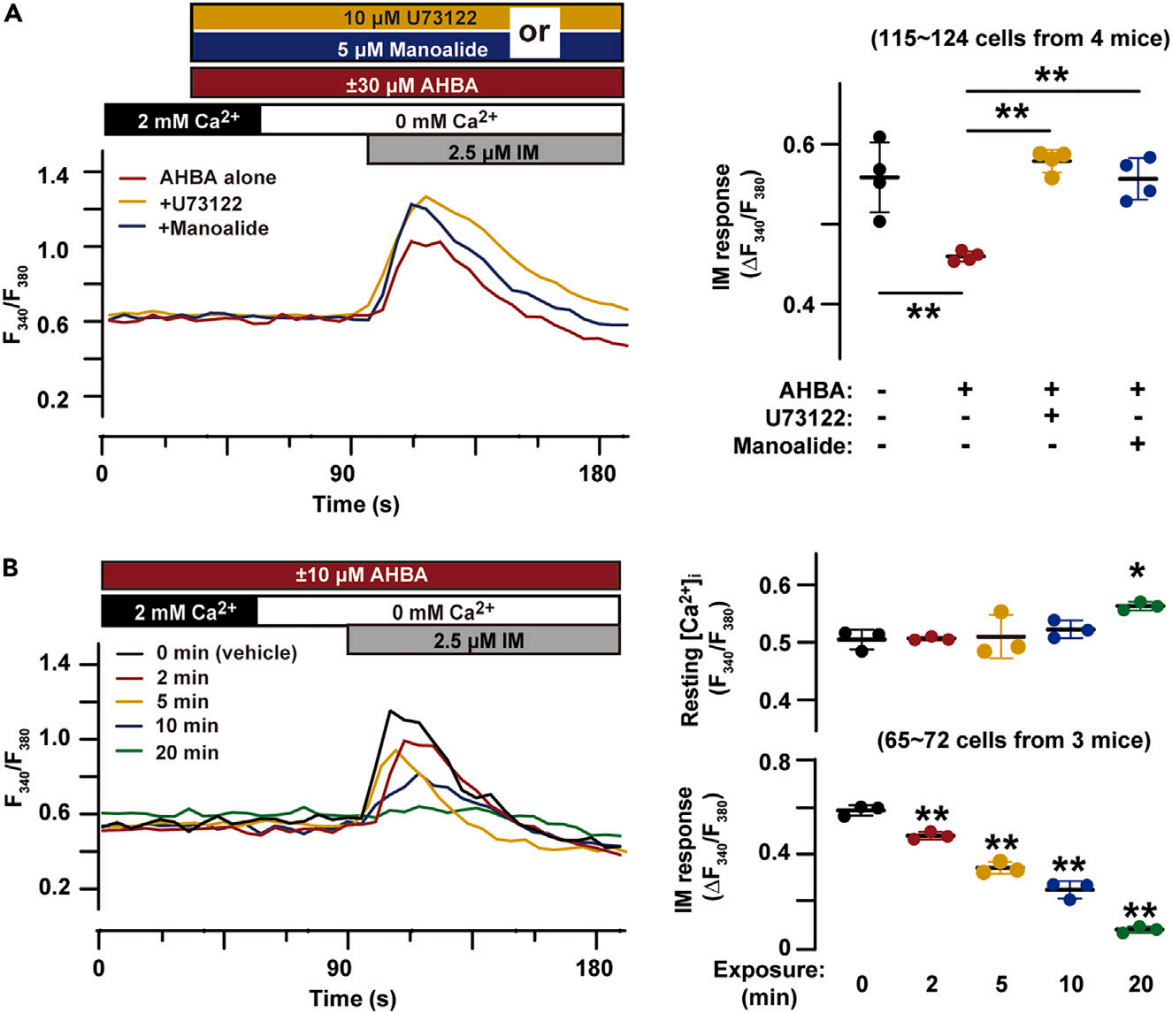
PLC and AHBA-induced Ca^2+^ store reduction (A) Effects of PLC inhibitors on AHBA-induced Ca^2+^ store reduction. The imaging traces show averaged time courses (n = 10 TGPMs in each group). The PLC inhibitors U73122 and manoalide were separately co-treated with AHBA. In the dot plot, Ca^2+^ responses evoked by ionomycin (IM) are statistically analyzed. (B) Exposure time-dependent effects of AHBA on Ca^2+^ stores. The imaging traces show the average time courses (n = 10 TGPMs in each group). TGPMs were treated with AHBA for various times (2–20 min) before ionomycin administration. In the dot plots, resting [Ca^2+^]_i_ at the final perfusion with the Ca^2+^-containing bathing solution and Ca^2+^ responses evoked by ionomycin are statistically analyzed. The data represent means ± SEM., and the numbers of cells and mice examined are shown in parentheses. Significant differences between groups indicated by bars (A) or from the vehicle-treated group (B) are marked with asterisks (∗p < 0.05 and ∗∗p < 0.01 in ANOVA and Dunnett’s test).

In contrast to ATP-induced Ca^2+^ release through IP_3_ receptor channels (Figure 1B), TGPMs exhibited no Ca^2+^ response to caffeine, an effective activator for ryanodine receptor channels (Figure S2A). This observation was consistent with the negligible expression of the *Ryr* subtype genes in TGPMs,^14^ indicating that IP_3_ receptors function as predominant Ca^2+^ release channels in TGPMs. Although IP_3_ receptor gating is dependent on both IP_3_ and Ca^2+^,^15^ short-term AHBA treatment reduced Ca^2+^ stores without affecting resting [Ca^2+^]_i_ in TGPMs (Figure 1A). To examine if AHBA dampened Ca^2+^ stores by facilitating steady-state IP_3_ generation, we used the phospholipase C (PLC) inhibitors U73122 and manoalide. Co-application of either the inhibitor diminished AHBA-induced reduction in store Ca^2+^ contents (Figure 2A). Moreover, the AHBA-induced reduction was clearly time-dependent; longer AHBA exposure resulted in more severe reduction in Ca^2+^ stores (Figure 2B). After a prolonged AHBA exposure (10 μM for > 20 min), intracellular stores were largely depleted, and the resting [Ca^2+^]_i_ became elevated in a Ca^2+^-containing bath solution. The elevated [Ca^2+^]_i_ was thought to be due to facilitated SOCE driven by the STIM-Orai activation.^16^

The previous observations suggested that AHBA facilitated IP_3_ generation by moderately enhancing “idling” PLC activity under resting conditions. The resulting enhanced IP_3_ generation might gradually stimulate Ca^2+^ leakage mediated by IP_3_ receptors, but did not trigger Ca^2+^ transients or waves, either of which requires the coincident activation of large numbers of IP_3_ receptors. In addition to IP_3_ liberation, PLC also generates diacylglyceroles (DAGs), which are endogenous activators of protein kinase C (PKC). However, the PKC inhibitor bisindolylmaleimide IV exerted no effect on AHBA-induced reduction in Ca^2+^ stores, suggesting that PKC did not affect store Ca^2+^-handling in TGPMs (Figure S2B).

### AHBA altered phospholipid metabolism

**Figure 3:**
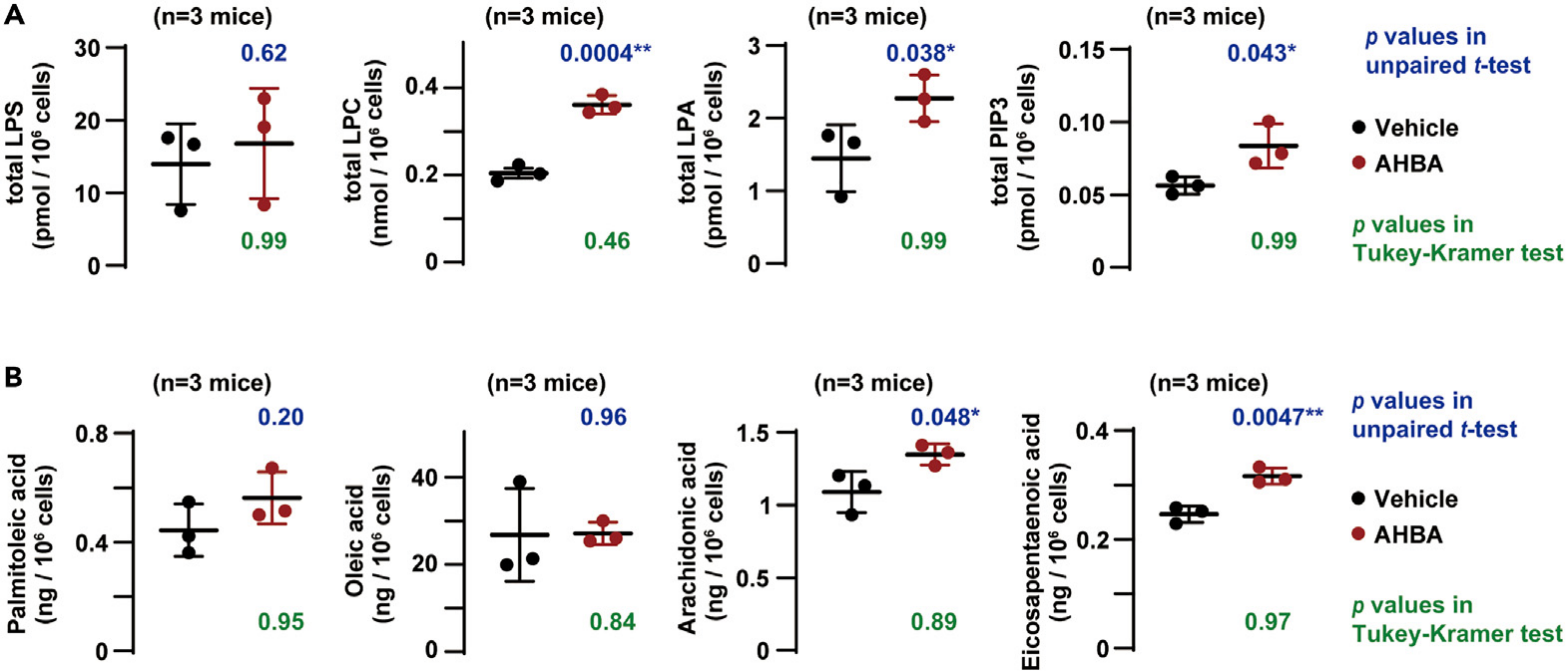
AHBA altered lipid metabolism (A) Notable observations in multi-phospholipid analysis. (B) Notable observations in eicosa/docosanoid analysis. The graphs focus on representative lipid metabolites with and without differences in cellular content between vehicle- and AHBA-treated TGPMs. The p values were calculated using the un-paired *t*-test and Tukey-Kramer test (∗p < 0.05 and ∗∗p < 0.01); the Turkey-Kramer test was performed in response to the reviewer’s request. The data represent means ± SEM., and the numbers of mice examined are shown in parentheses. The detailed data obtained from the lipidome analyses are summarized in Tables S1 and S2.

We next examined AHBA-induced effects on lipid metabolism. Total lipid extracts prepared from TGPMs treated with or without AHBA (10 μM for 5 min) were subjected to outsourced lipid metabolomic analysis. As notable observations in the multi-phospholipid analysis, lysophosphatidylcholine (LPC), LPA and phosphatidylinositol triphosphate (PIP_3_) contents were tended to increase in AHBA-treated TGPMs when compared with control cells (Figure 3A; Table S1). The elevated LPC content probably reflected the specific hydrolysis of PCs, because AHBA treatment did not affect other lyso-phospholipid species. The eicosa/docosanoid analysis likely suggested that AHBA treatment increased contents of some PUFAs such as arachidonic and eicosapentaenoic acids without affecting non-PUFA contents (Figure 3B and Table S2). It was also predicted that the generation of thromboxane B2 (TXB2), a non-enzymatic metabolite of thromboxane A2 (TXA2), was stimulated in AHBA-treated TGPMs (Table S2). This observation, together with immunochemical TXB2 measurements (Figure 7), suggested that AHBA treatment facilitated TXA2 synthesis.

Of the N-phos substrates reported thus far,^5^^,^^6^^,^^7^ LPAs significantly accumulated in AHBA-treated cells. Therefore, the conversion of LPAs to MAGs may be mediated predominantly by N-phos in TGPMs. On the other hand, several possibilities could be drawn from the AHBA-induced shifts in lipid metabolite; however, one reasonable explanation is as follows. The increased LPC and PUFA contents suggested that AHBA may stimulate phospholipase A2 (PLA2), while the elevated PIP_3_ content suggested that phosphoinositide 3-kinase (PI3K) may be activated by AHBA. Furthermore, we focused on the LPA accumulation and TXA2 induction in AHBA-treated TGPMs, because both of the metabolites act as bioactive autacoids.

### AHBA autocrine-activated LPA receptors

**Figure 4:**
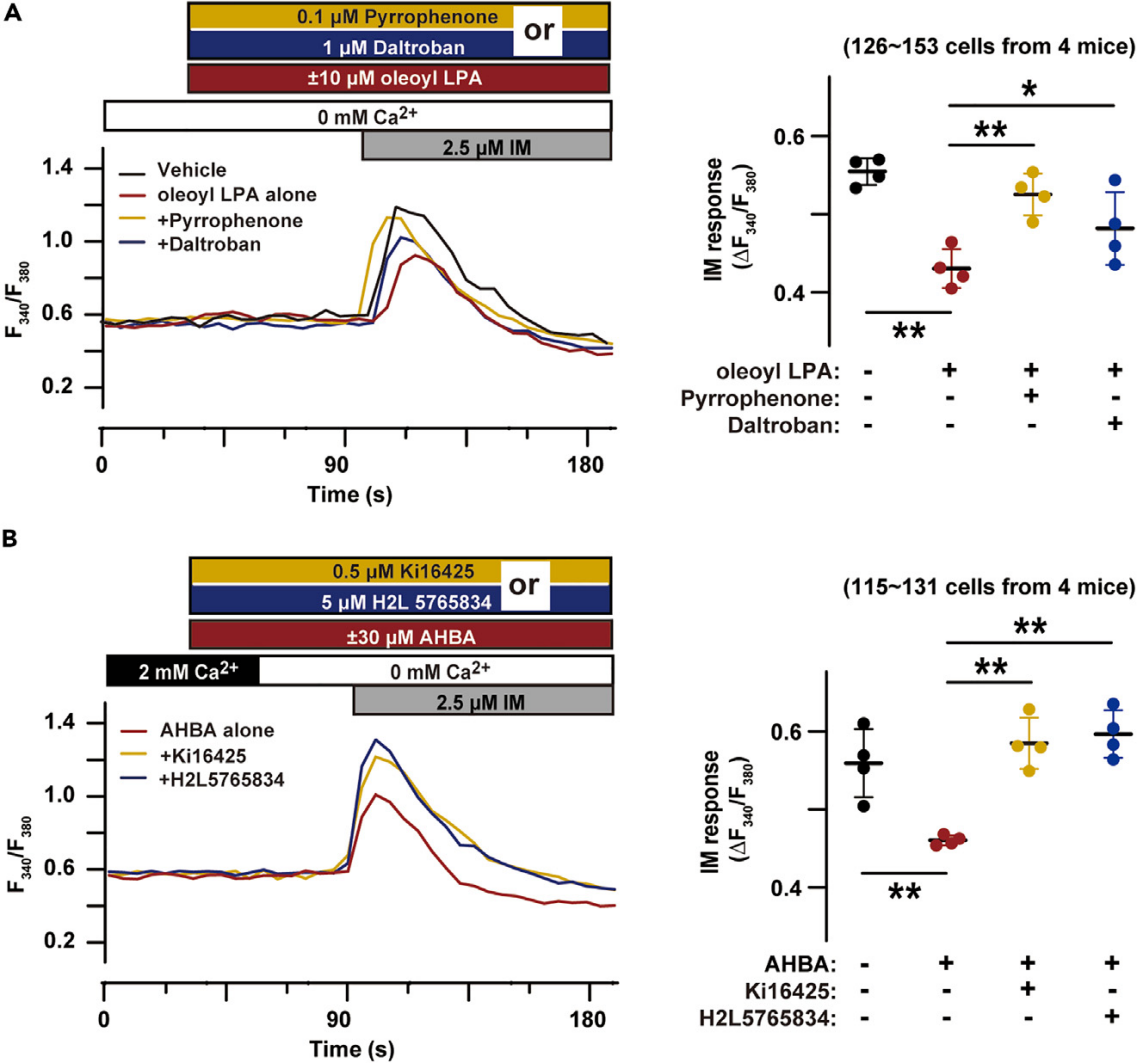
LPA receptors and AHBA-induced Ca^2+^ store reduction (A) LPA-induced Ca^2+^ store reduction and TXA2 signaling. The imaging traces show the average time courses (n = 10 TGPMs in each group). TGPMs were treated with or without oleoyl-LPA and store Ca^2+^ contents were examined by ionomycin (IM). LPA-induced effects were further examined by the co-treatment of the cPLA2 inhibitor pyrrophenone or the TXA2 receptor antagonist daltroban. (B) AHBA-induced Ca^2+^ store reduction and LPA signaling. The LPA antagonist Ki16425 or H2L5765834 was co-treated with AHBA before ionomycin application, and the imaging traces show the average time courses (n = 10 TGPMs in each group). In the dot plots, ionomycin-evoked Ca^2+^ responses are statistically analyzed. The data represent means ± SEM., and the numbers of cells and mice examined are shown in parentheses. Statistical differences between groups marked with lines are marked with asterisks (∗p < 0.05 and ∗∗p < 0.01 in ANOVA and Dunnett’s test).

Gene expression profiles in a public database and our observations in reverse transcription polymerase chain reaction (RT-PCR) and western blot analyses indicated that the LPA receptor subtype genes *Lpar1* and *Lpar5* were weakly expressed in TGPMs (Figure S3). LPAR1 and LPAR5 subtypes are known to potentially couple to the guanine nucleotide-binding proteins Gq, Gi/o, and G_12/13_.^17^ The application of oleoyl-LPA at a submaximal dose induced no Ca^2+^ transient in TGPMs (Figure 4A), in contrast to definitive Ca^2+^ transients triggered by ATP-induced P2Y receptor activation (Figure 1B). This discrepancy was likely due to contrasting receptor densities and G-protein-coupling orientations; *Lpar* mRNA content was roughly estimated to be at least an order magnitude less than *P2ry* mRNA content (Figure S3), and LPA receptors did not efficiently couple to Gq in TGPMs as explained further.

Similar to AHBA treatment, oleoyl-LPA clearly reduced ionomycin-induced Ca^2+^ release within a few minutes in TGPMs (Figure 4A). However, unexpectedly, the oleoyl-LPA-induced reduction in Ca^2+^ stores was largely attenuated by the PLA2 inhibitor pyrrophenone and the TXA2 receptor antagonist daltroban (Figure 4A). Therefore, TXA2 production and signaling may essentially contribute to the reduction induced by LPA receptor activation, and it was unlikely that LPA receptor activation directly induced Gq-mediated PLC stimulation.

Of the LPA receptor antagonists that have been variedly developed, Ki16425 preferentially inhibits LPAR1/3, and H2L5765834 selectively blocks LPAR1/3/5. When either antagonist was co-treated, AHBA did not reduce ionomycin-induced Ca^2+^ release (Figure 4B), suggesting that AHBA inhibited the conversion of LPA to MAG to increase intracellular LPA content and thus induced autocrine activation of LPA receptors toward Ca^2+^ store reduction. Taken together with the effects of the TXA2 signaling inhibitors, co-activation of LPA and TXA2 receptors likely underlay AHBA-induced reduction in store Ca^2+^ content in TGPMs.

### AHBA facilitated kinase signaling

**Figure 5:**
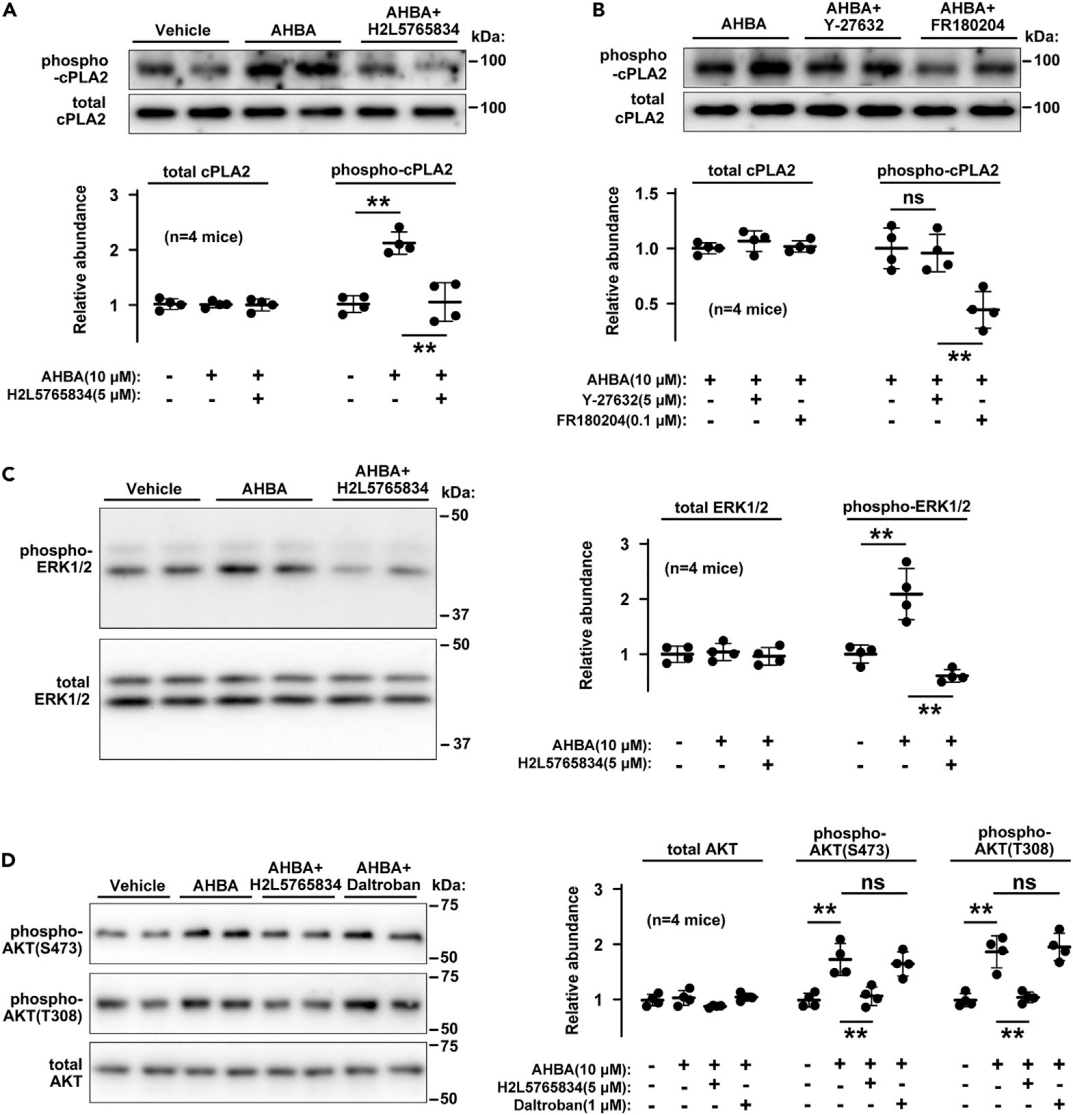
AHBA activated kinase signaling (A) cPLA2 phosphorylation and AHBA-induced LPA receptor activation. Total cPLA2 and its phosphorylated form were examined in TGPMs treated with the indicated inhibitors. (B) cPLA2 phosphorylation and AHBA-induced ERK activation. Total cPLA2 and its phosphorylated form were examined in TGPMs treated with the indicated inhibitors. (C) ERK phosphorylation and AHBA-induced LPA receptor activation. Total ERK and its phosphorylated form were examined in TGPMs treated with the indicated inhibitors. (D) AKT phosphorylation and AHBA-induced LPA receptor activation. Total AKT and its Thr308-and Ser473-phosphorylated forms were examined in TGPMs treated with the indicated inhibitors. The inhibitors used are the LPA receptor blocker H2L5765834, the TXA2 receptor blocker daltroban, the ERK inhibitor FR 180204, the ROCK inhibitor Y-27632, and the PI3K inhibitor TG100-116. Total cell lysates were prepared from TGPMs treated with the combination of inhibitors for 20 min and subjected to immune-blot analysis. The resulting immune-signals were captured as shown in the representative digital images and quantitatively analyzed as presented in the plot graphs. The relative abundance is related to the average level of the right-side group in each graph. The data represent means ± SEM., and the mice examined are shown in parentheses. Significant differences between the groups indicated by bars are statistically examined (ns: not significant, ∗p < 0.05 and ∗∗p < 0.01 in ANOVA and Sidak’s test).

Of the divergent PLA2 subtypes, cytosolic phospholipase A2a (cPLA2, also known as PLA2G4A) often contributes to prostanoid and eicosanoid production in response to various stimuli.^18^ Extracellular signal-regulated kinase (ERK) and Rho-associated protein kinase (ROCK) are stimulated downstream of Gi/o and G_12/13_, respectively,^19^^,^^20^ and both kinases can activate cPLA2 by phosphorylating Ser505.^21^^,^^22^ In western blot analysis of total cell lysates prepared from TGPMs, the signal intensity of phospho-cPLA2 was markedly increased in response to AHBA treatment (10 μM for 20 min). This increase was inhibited by the co-application of H2L5765834 (Figure 5A). In addition, AHBA-induced cPLA2 phosphorylation was disrupted by the ERK inhibitor FR180204, while the ROCK inhibitor Y-27632 had no effect (Figure 5B). Therefore, AHBA-induced LPA receptor activation probably stimulated ERK-mediated cPLA2 phosphorylation, but was unlikely to efficiently facilitate G_12/13_-ROCK signaling in TGPMs.

ERK is activated by phosphorylation at Thr183 and Tyr185 by mitogen-activated protein kinase kinase (MEK) downstream of Gi/o activation.^19^ AHBA treatment elevated phospho-ERK content, and this elevation was inhibited by H2L5765834 (Figure 5C). Therefore, cPLA2 was probably activated downstream of Gi/o-ERK signaling resulting from LPA receptor activation in AHBA-treated TGPMs. The activated cPLA2 was thought to selectively react with PCs to generate lyso-PCs and PUFAs, because PCs are preferable substrates for cPLA2.^23^ Furthermore, it has been also reported that PUFAs generated by cPLA2 activation are predominantly converted into TXA2 in macrophages.^24^

Downstream of Gi/o signaling, in parallel with the ERK activation, PI3K is often stimulated to generate PIP_3_-rich membrane compartments, where AKT kinase (protein kinase B) is assembled and activated.^25^ The active form of AKT contains phospho-Thr308 and phospho-Ser473, which are separately phosphorylated by phosphoinositide-dependent kinase 1 (PDK1) and mammalian target of rapamycin complex 2 (mTORC2), respectively. PDK1 activation likely requires ERK activity; ERK phosphorylates and activates ribosomal S6 kinase-2 (RSK2), and the activated RSK2 then recruits and activates PDK1 in a COS7 expression system.^26^ In AHBA-treated TGPMs, AKT phosphorylation was elevated at both sites, and this elevation was abolished by H2L5765834 (Figure 5D) and the PI3K inhibitor TG100-115 (Figure S4A). Therefore, LPA receptor activation seemed to sequentially induce PI3K and AKT activation in AHBA-treated TGPMs. Moreover, FR180204 significantly inhibited PDK1-dependent Thr308 phosphorylation but exerted no obvious effect on mTORC2-dependent Ser473 phosphorylation (Figure S4A), suggesting that ERK also contributed to PDK1-mediated AKT activation in TGPMs. On the other hand, as expected, the mTORC1/2 inhibitor torin 1 disrupted AHBA-induced Ser473 phosphorylation without affecting T308 phosphorylation (Figure S4B).

### AHBA autocrine-activated TXA2 receptors

**Figure 6:**
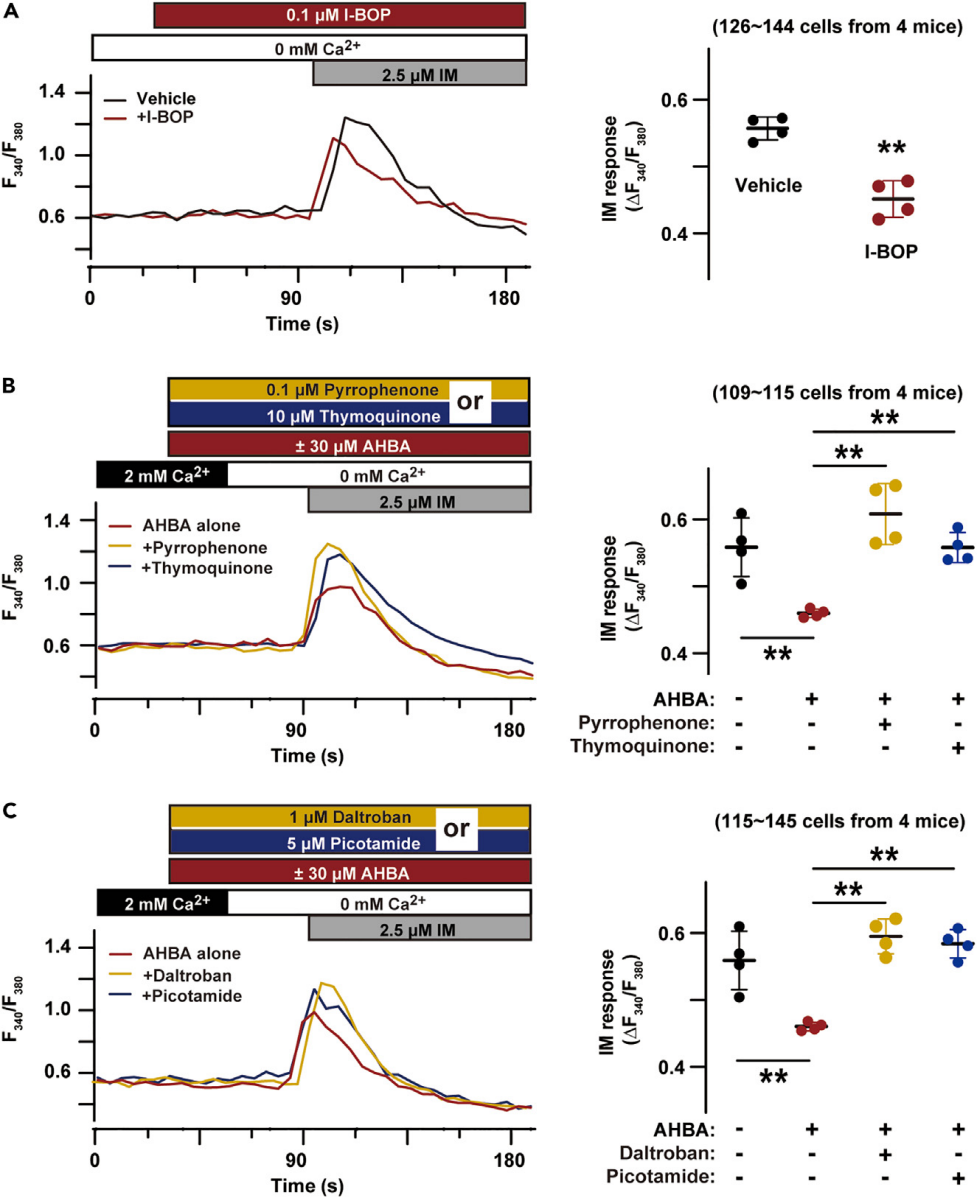
TXA2 receptors and AHBA-induced Ca^2+^ store reduction (A) TXA2 receptor activation and Ca^2+^ store reduction. TGPMs were treated with or without the TXA2 receptor agonist I-BOP before ionomycin (IM) application, and the Fura-2 imaging traces show the average time courses (n = 10 TGPMs in each group). (B) TXA2 synthesis and AHBA-induced Ca^2+^ store reduction. The cPLA2 inhibitor pyrrophenone or the COX/LOX inhibitor thymoquione was co-treated with AHBA, and the imaging traces show the average time courses (n = 10 TGPMs in each group). (C) TXA2 receptor activation and AHBA-induced Ca^2+^ store reduction. The TXA2 receptor antagonists daltroban and picotamide were separately co-treated with AHBA, and the imaging traces show the average time courses (n = 10 TGPMs in each group). Ionomycin-induced responses are statistically examined in the plot graphs. The data represent means ± SEM. and the numbers of cells and mice examined are shown in parentheses. Statistical differences between the two groups in (A) and the groups indicated by bars in (B and C) are marked with asterisks (∗∗p < 0.01 in *t*-test [A] and ANOVA and Dunnett’s test [B and C]).

TXA2 primarily functions as an auto/paracrine mediator to activate TXA2 receptors coupling with Gq.^18^ In gene expression and western blot analyses, the TXA2 receptor gene *Tbx2r* was marginally active in TGPMs, and the estimated mRNA content was roughly similar to that of *Lpar* mRNAs (Figure S3). In Ca^2+^ imaging, the TXA2 receptor agonist I-BOP did not induce Ca^2+^ transients but clearly reduced store Ca^2+^ content in TGPMs (Figure 6A). The PLA2 inhibitor pyrrophenone and the COX/LOX inhibitors licofelone and thymoquinone disrupted AHBA-induced reduction in Ca^2+^ stores (Figures 6B and S2C). Furthermore, the TXA2 receptor antagonists daltroban and picotamide also abolished the AHBA-induced reduction in Ca^2+^ store (Figure 6C). Therefore, TXA2 generation and TXA2 receptor activation seemed to contribute to the AHBA-induced effect on TGPMs. TXA2 receptor activation is supposed to stimulate Gq and its downstream effector PLCβ.^23^

### AHBA facilitated TXA2 and IP_3_ generation

**Figure 7:**
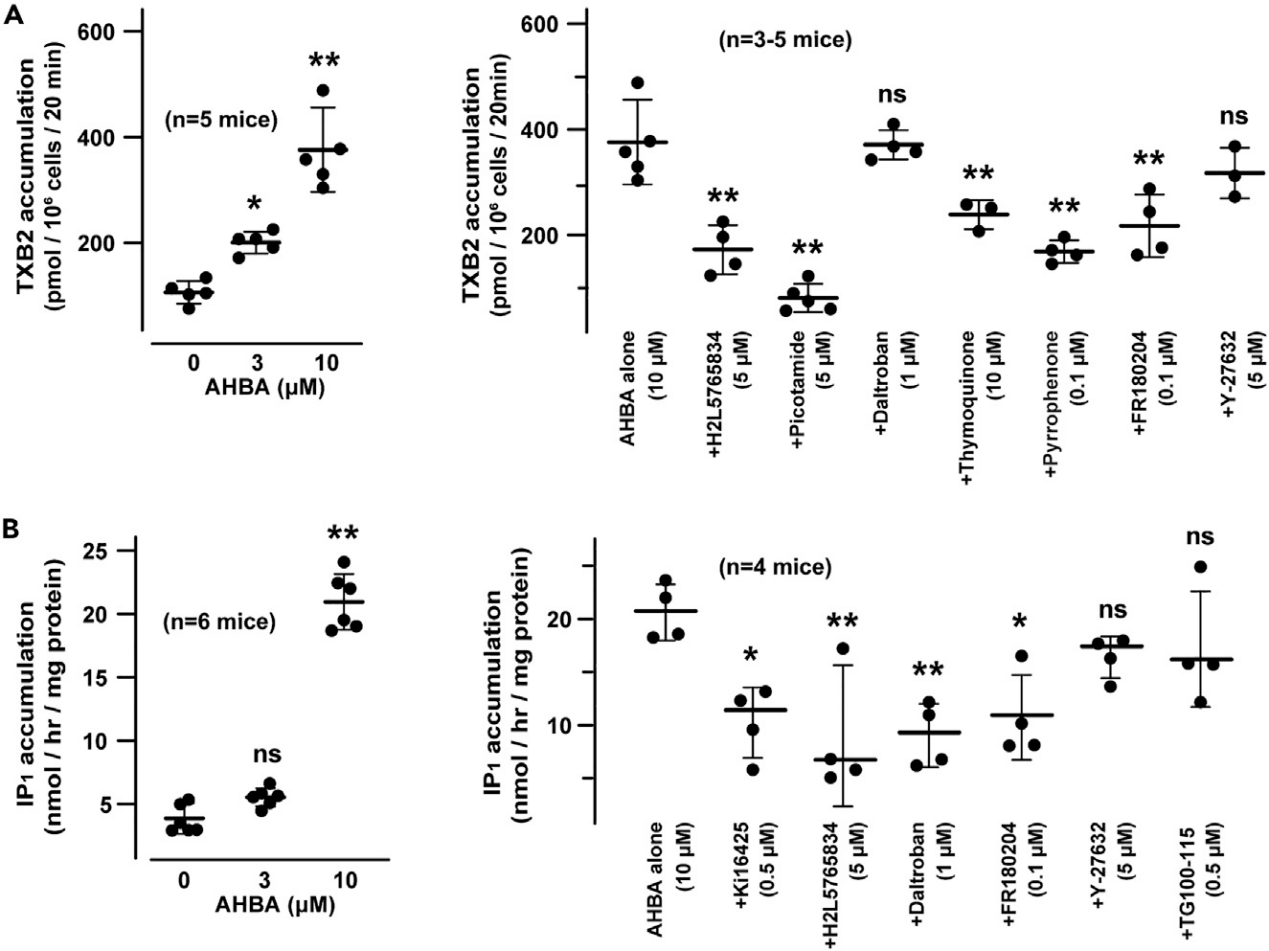
AHBA facilitated IP_3_ and TXA2 generation (A) AHBA-induced TXB2 accumulation. TGPMs were treated with or without AHBA for 20 min, and TXB2 contents in culture supernatants were immunochemically quantified (left graph). The AHBA-induced TXB2 accumulation was pharmacologically examined using the indicated inhibitors (right panel). (B) AHBA-induced intracellular IP_1_ accumulation. TGPMs were treated with or without AHBA for 20 min, and IP_1_ contents were immunochemically quantified (left graph). The AHBA-induced IP_1_ accumulation was pharmacologically examined using the indicated inhibitors (right panel). The inhibitors used are the LPA receptor antagonists Ki16425 and H2L5765834, the TXA2 receptor antagonists picotamide and daltroban, the COX/LOX inhibitor thymoquinone, the cPLA2 inhibitor pyrrophenone, the ERK inhibitor FR180204, the ROCK inhibitor Y-27632, and the PI3K inhibitor TG100-115. The data represent means ± SEM., and the numbers of mice examined are shown in parentheses. Significant differences from the vehicle-treated groups (left graphs) or AHBA-treated groups (right graphs) are marked with asterisks (∗p < 0.05 and ∗∗p < 0.01 in ANOVA and Dunnett’s test).

To confirm the predicted induction of TXA2 generation in AHBA-treated TGPMs (Table S2), we used an enzyme-linked immunosorbent assay (ELISA) kit to quantify TXB2 released into cultured supernatants. AHBA treatment (10 μM for 20 min) markedly elevated TXB2 content, indicating that TXA2 production was enhanced in AHBA-treated TGPMs (Figure 7A). TXB2 contents were also increased in TGPMs treated with the N-phos inhibitor oxaprozin and the dual inhibitor ebselen (Figure S5A). AHBA-induced TXB2 accumulation was inhibited by co-treated H2L5765834, thymoquinone, pyrrophenone, or FR180204, supporting the contribution of LPA receptors, ERK, cPLA2, and COX to the facilitated TXA2 production. Although picotamide and daltroban are both TXA2 receptor antagonists, picotamide is known to also act as a thromboxane synthase inhibitor and thus seemed to inhibit the AHBA-induced enhancement.

We next examined AHBA-induced effects on IP_3_ generation using a commercial kit for quantifying intracellular IP_1_ as a surrogate for IP_3_ generated during incubation period. AHBA treatment (10 μM for 20 min) significantly elevated IP_1_ content in TGPMs, indicating AHBA-induced facilitation of IP_3_ generation (Figure 7B). IP_1_ contents were also increased in TGPMs treated with oxaprozin and ebselen (Figure S5B). The AHBA-induced IP_1_ accumulation was inhibited by co-treated Ki16425, H2L5765834, and daltroban (Figure 7B), consistent with their effects on AHBA-induced reduction in Ca^2+^ stores (Figures 4 and 6). Of the kinase inhibitors tested, FR180204 significantly suppressed the AHBA-induced IP_1_ accumulation likely due to disruption of ERK-mediated cPLA2 activation for TXA2 synthesis. Therefore, AHBA likely facilitated steady-state IP_3_ production in a manner that was dependent on sequential activations of LPA receptors, ERK, cPLA2, TXA2 receptors, and PLC in TGPMs.

### AHBA attenuated macrophage functions

**Figure 8:**
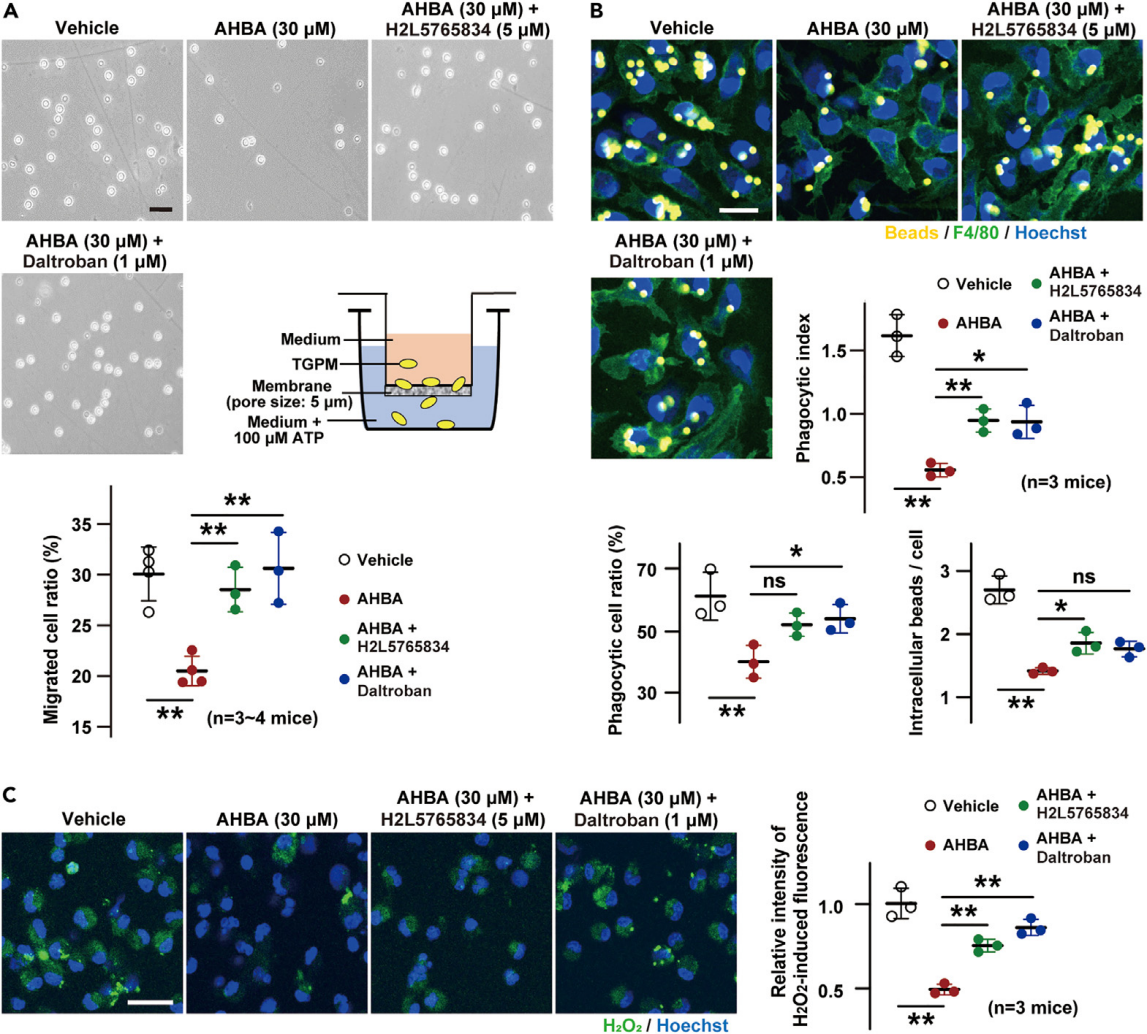
AHBA attenuated macrophage functions (A) AHBA-induced inhibition of chemotaxis. In the migration assay schematically illustrated; the medium contained the indicated combinations of the inhibitors. After incubation, TGPMs that migrated to the bottom chamber were observed (image panels, scale bar = 25 μm), and the migration capacity was statistically analyzed (lower graph). (B) AHBA-induced inhibition of phagocytosis. Phagocytosed beads in TGPMs were captured after incubation (image panels, scale bar = 25 μm), and phagocytotic cell ratio and capacity were statistically analyzed. Phagocytic index = (mean intracellular beads in phagocytic cell)×(phagocytic cell ratio). (C) AHBA-induced inhibition of H_2_O_2_ production. TGPMs were incubated with the indicated combinations of the inhibitors and the H_2_O_2_-reactive indicator AbGreen, and then examined under a fluorescence microscopy. H_2_O_2_-reacted fluorescence generated in TGPMs was captured (image panels, scale bar = 25 μm), and the fluorescence intensity are statistically analyzed in the graph. The numbers of mice examined are shown in parentheses, and the data represent means ± SEM. Statistical differences between groups indicated by bars are marked with asterisks (∗p < 0.05, ∗∗p < 0.01 and ns, not significant in ANOVA and Dunnett’s test).

AHBA had no severe toxic effect on TGPMs at less than 100 μM during 24-h incubation (Figure S5C). We next examined the effects of AHBA (30 μM) on chemotaxis, phagocytosis, and H_2_O_2_ generation, all of which are fundamental macrophage functions. In migration across a membrane equipped with micropores during 4 h, TGPM chemotaxis toward ATP was inhibited by AHBA (Figure 8A). This inhibitory effect was almost eliminated by co-treated H2L5765834 and daltroban. AHBA also inhibited migration to C-C motif chemokine ligand 2 (CCL2), but did not affect chemotaxis to the bacterial outer membrane component lipopolysaccharide (LPS) (Figure S5D). The results suggested that AHBA can broadly attenuate migration signaling initiated by activation of G-protein coupled receptors as the sensors of chemical gradients.

In the phagocytosis assay monitoring the uptake of fluorescence-labeled microbeads for 3 h, AHBA significantly inhibited both phagocytic cell rates and capacities, thus leading to marked reduction in phagocytic index (Figure 8B). Co-treated H2L5765834 and daltroban considerably restored the phagocytic index reduced by AHBA. In the imaging assay using an H_2_O_2_-sensitive indicator, AHBA inhibited the accumulation of fluorescence-positive dye which was produced by reacting with H_2_O_2_ during 3-h incubation, indicating that NADPH oxidase 2 (NOX2) activity was attenuated in AHBA-treated TGPMs (Figure 8C). This AHBA-induced attenuation was almost abrogated by co-treated H2L5765834 and daltroban.

### AHBA attenuated macrophage M1 polarization

**Figure 9:**
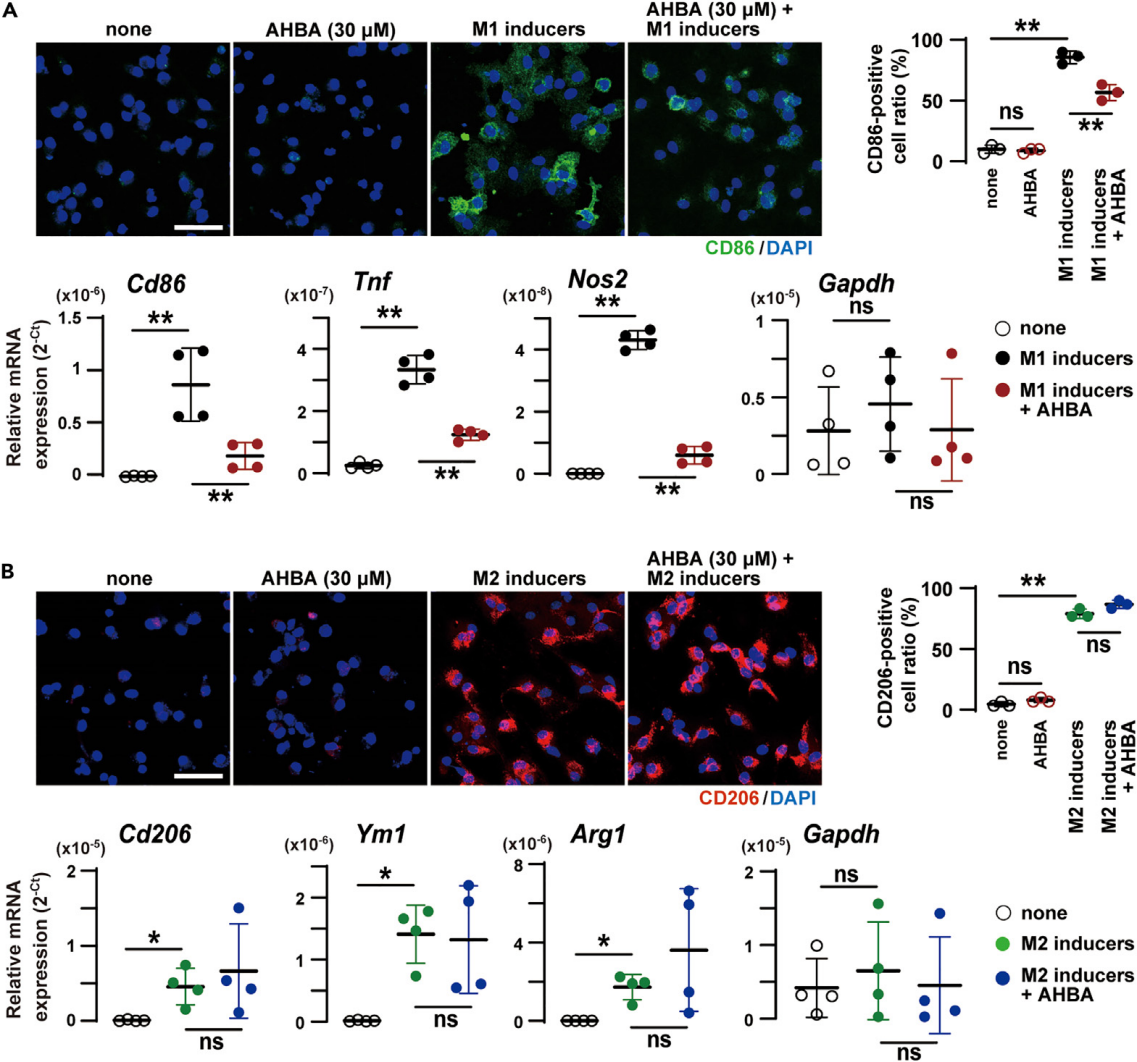
AHBA attenuated M1 polarization (A) Effects of AHBA on M1 polarization. The effects of co-applicated AHBA were examined in BMDMs stimulated with or without the M1-polarizing mediators. The resulting cells were immunostained for analyzing CD86-positive cell ratios (upper images and graph), and also subjected to RT-PCR analysis for the M1 marker gene expression (lower graphs). (B) Effects of AHBA on M2 polarization. The effects of co-applicated AHBA were examined in BMDMs treated with or without the M2-polarizing mediators. The treated cells were immunostained for analyzing CD206-positive cell ratios (upper images and graph), and also subjected to RT-PCR analysis for the M2 marker gene expression (lower graphs). In the graphs, the numbers of mice examined are shown in parentheses, and the data represent means ± SEM. Statistical differences between groups indicated by bars are marked by asterisks (∗p < 0.05, ∗∗p < 0.01 and ns, not significant in ANOVA and Tukey-Kramer test). Scale bars in the fluorescence images, 25 μm.

By established mediator treatments for 24 h, primary-cultured marrow-derived macrophages (BMDMs) can be polarized to M1-and M2-like macrophages.^27^ BMDMs largely became positive for the M1 marker CD86 in response to the M1 inducers LPS and interferon-β (IFN-β), and co-treated AHBA (30 μM) remarkably reduced the appearance of CD86-positive cells (Figure 9A). RT-PCR analysis detected that the representative M1-marker genes *Cd86*, *Tnf* for tumor necrosis factor alpha (TNF-α) and *Nos2* for inducible nitric oxide synthase 2 were highly activated after the M1-polarizing treatment, and the upregulations were largely attenuated by co-treated AHBA. On the other hand, BMDMs turned positive for the M2 marker CD206 in response to the M2 inducers interleukin-4 (IL-4) and IL-13, and co-treated AHBA did not affect CD206-positive cell ratios (Figure 9B). The M2-marker genes *Cd206*, *Ym1* for chitinase 3-like 3 and *Arg1* for arginase 1 were transcriptionally upregulated after the M2-inducing treatment, and co-treated AHBA did not alter the M2-maker gene expression. Therefore, AHBA seemed to attenuate M1 polarization without affecting M2 polarization in BMDMs. Both IL-4 and IL-13 react to the receptor complexes containing the IL-4 receptor α, and activate overlapping signaling systems so-called JAK-STAT pathways, in which JAK kinase phosphorylates and activates the signal transducer and activator of transcription (STAT) proteins.^28^ Our observation suggested that AHBA exerted no obvious effects on JAK-STAT signaling.

The powerful M1 inducer LPS stimulates cell-surface toll-like receptors (TLRs) and activates several signaling pathways, leading to induction of inflammation-related gene products in macrophages. Of the LPS-inducible M1 polarization-related genes previously reported,^26^ 10 typical inflammation-related genes were selected for our RT-PCR analysis (Figure S8). For example, mRNAs derived from the *Tnf* and *Il1b* for IL-1β were obviously induced by the LPS treatment for 4 h, and these LPS-induced effects were largely inhibited by co-treated AHBA (30 μM). The AHBA-induced effects were occasionally interrupted by co-treated H2L5765834 and daltroban; the interruption efficiencies of the receptor blockers were highly varied among the genes. Consistent with the RT-PCR data, ELISA quantification showed that LPS-induced TNF-α secretion was moderately inhibited by AHBA, and that the receptor blockers attenuated the AHBA-induced inhibition (Figure S5E). The observations likely suggested that AHBA broadly inhibited M1-related gene expression and thus attenuated M1 polarization.

## Discussion

In this study, we examined the mechanism underlying the AHBA-induced reduction in store Ca^2+^ content in TGPMs. Based on the data obtained, we propose an N-phos-mediated functional link between steady-state lipid metabolism and autocrine signaling, and the proposed scheme, together with a simplified PC turnover cycle, is illustrated in Figure S7. Leuiller et al. reported that N-phos likely converts LPAs to MAGs in hepatocytes based on lipidomic data from N-phos-deficient knock-in rats.^8^ In accordance with their conclusion, AHBA-induced alterations in lipid profiles suggest that N-phos is also mainly responsible for metabolizing LPAs to MAGs in TGPMs (Figure 3). Our proposed scheme has the following sequential events; (1) AHBA inhibits the conversion of LPAs to MAGs, (2) LPAs thus accumulate and weakly stimulate intrinsic LPA receptors coupling to Gi/o, (3) downstream of Gi/o activation, cPLA2 is stimulated by ERK-mediated phosphorylation, and AKT is also stimulated in the PI3K-dependent manner, (4) Activated cPLA2 then generates excessive LPCs and PUFAs, (5) accumulated PUFAs are predominantly converted to TXA2, which is further transformed to inactive TXB2, (6) TXA2 stimulates intrinsic TXA2 receptors, (7) TXA2 receptor activation potentiates PLCβ activity through Gq stimulation to stimulate steady-state IP_3_ generation, (8) finally, increased IP_3_ production gradually facilitates IP_3_ receptor-mediated Ca^2+^ leakage to reduce store Ca^2+^ content. Our observations in AHBA-treated TGPMs suggest that TXA2 receptor activation requires LPA receptor activation (Figure 6), and both TXA2 and LPA receptor antagonists had similar inhibitory effects on the AHBA-induced PLC activation and reduction in Ca^2+^ stores (Figures 4 and 7). Therefore, TXA2 receptors seem to be mainly responsible for AHBA-induced PLC activation, while the direct contribution of LPA receptors may be negligible. AHBA primarily inhibits dephosphorylation of LPAs, but may fine-readjust a functional link between phospholipid turnover, mediator synthesis, and autocrine signaling in TGPMs. Since LPA receptor subtypes are differentially expressed in various cell types,^17^ N-phos inhibition may generally increase intracellular LPA contents for autocrine-activation of endogenous LPA receptors, leading to the modulation of various intracellular signaling in different cell types. However, the predicted modulations may be commonly accompanied by PLC activation because AHBA reduced store Ca^2+^ contents in several non-excitable cell types tested (Figure S1B).

We demonstrated that AHBA attenuated the macrophage-related functions in TGPMs (Figure 8). Downstream of AHBA-induced LPA receptor activation, ERK and AKT pathways and TXA2 receptor-mediated store Ca^2+^ leakage are probably stimulated as mentioned previously. All of the proposed effects would be blocked by H2L5765834, while daltroban would interrupt PLC activation and following Ca^2+^ leakage from stores. Both PI turnover and store Ca^2+^ handling influence a wide variety of cellular responses. For example, migration and phagocytosis require PLC activity and Ca^2+^-dependent processes for controlling membrane dynamics,^29^^,^^30^ and H_2_O_2_-producing NOX2 activity is stimulated by Ca^2+^ in cardiac myocytes.^31^ The AHBA-induced inhibitions of chemotaxis, phagocytosis, and H_2_O_2_ generation were largely attenuated by both the LPA and TXA2 receptor blockers at similar potencies (Figure 8), suggesting that the inhibitions are mainly due to PLC activation and/or deranged Ca^2+^ stores in AHBA-treated TGPMs.

AHBA inhibited LPS-induced gene expression, likely leading to attenuation of *in vitro* M1 polarization (Figures S6 and 9). LPS-induced TLR activation stimulates several Ser/Thr-kinase pathways to activate the transcription factors NF-κB (NF-κB), activator protein 1 (AP-1) and interferon regulatory factors 3 and 5 (IRF3/5), facilitating transcription of inflammation-related genes.^32^^,^^33^ Activation of NF-κB and AP-1 is regulated by intracellular Ca^2+^ (ref. 34,35). On the other hand, the PI3K/AKT pathway converges the control of inflammatory responses by functioning for the negative regulation of NF-κB-mediated gene expression in macrophages.^36^ In fact, AKT overexpression impairs inflammation-related responses in macrophages, while inflammatory lesions are exacerbated in AKT-deficient mice.^37^ Furthermore, in response to LPS-evoked TLR activation, the Tyr-kinase Syk and its downstream effector PLCγ are also activated and act as the important signaling regulators.^38^ Store Ca^2+^ release predicted in the Sky-PLCγ pathway is likely interfered by AHBA-induced activation of store Ca^2+^ leakage. Therefore, activated AKT signaling and store Ca^2+^ leakage may mainly contribute to the inhibition of LPS-induced inflammation-related gene activation in AHBA-treated TGPMs. NF-κB, AP-1, and IRF3/5 differentially boost transcription depending on the gene, and it may be reflected by the observations that AHBA and receptor blockers exerted divergent effects on the modulation of the LPS-inducible gene expression.

Macrophages are closely associated with tissue inflammation, and our observations suggest that N-phos inhibition broadly attenuates inflammatory functions in macrophages. Therefore, we propose that N-phos is a surefire target for developing anti-inflammatory drugs. In previous studies on sEH, the dual inhibitors that block both N-phos and C-EH were often used, and the role of C-EH in the regulation of inflammation was emphasized. Our present observations suggest the possibility that the pathophysiological role of C-EH might have been overestimated. After all, it is thus important to re-examine the anti-inflammatory role of sEH inhibitors by clearly dissecting the distinct effects induced by N-phos and C-EH inhibitions.

### Limitation of the study

The major limitation of this study is only based on assessments using chemical tools. To further confirm our conclusion, it is necessary to prepare N-phos-deficient knock-in macrophage models for comparing pharmacological effects between the mutant and wild-type cells.

## STAR★Methods

### Key resources table

**Table:** 

REAGENT or RESOURCE	SOURCE	IDENTIFIER
**Antibodies**		

Phospho-cPLA2 (Ser505)	Cell Signaling Technology	Cat#2831S; RRID: AB_2164442
cPLA2	Cell Signaling Technology	Cat#2832S; RRID: AB_2164445
Phospho-p44/42 MAPK (Erk1/2) (Thr202/Tyr204) (E10) Mouse mAb	Cell Signaling Technology	Cat#9106; RRID:AB_331768
p44/42 MAPK (Erk1/2)	Cell Signaling Technology	Cat#9102; RRID:AB_330744
Phospho-AKT(Ser473)	Cell Signaling Technology	Cat#9271; RRID:AB_329825
Phospho-Akt (Thr308) (C31E5E) Rabbit mAb	Cell Signaling Technology	Cat#2965; RRID:AB_2255933
AKT	Cell Signaling Technology	Cat#9272; RRID:AB_329827
Polyclonal Goat Anti-Rabbit Immunoglobulins/HRP	Agilent Technologies	Cat#P0448; RRID:AB_2617138
Polyclonal Rabbit Anti-Mouse Immunoglobulins/HRP	Agilent Technologies	Cat#P0260; RRID:AB_2636929
FITC anti-mouse F4/80 Antibody	Biolegend	Cat#123108; RRID:AB_893502
Purified anti-mouse CD86 Antibody	BioLegend	Cat#105102; RRID:AB_313155
Purified anti-mouse CD206 (MMR) Antibody	BioLegend	Cat#141701; RRID: AB_10900263
LPA receptor 5 (GPR92)	Merck	Cat#ABT114; NP_065133
TXA2R (G-2)	Santa Cruz Biotechnology	Cat#SC-515033; RRID:AB_2847878

**Biological samples**		

Mouse peritoneal macrophages; C57BL/6J	Institute of Medical Science, University of Tokyo	N/A
Mouse bone marrow derived macrophages; C57BL/6J	Institute of Medical Science, University of Tokyo	N/A
Mouse embryonic fibroblasts (MEF); C57BL/6J	Institute of Medical Science, University of Tokyo	N/A
Mouse Hepatocyte; C57BL/6J	Institute of Medical Science, University of Tokyo	N/A

**Chemicals, peptides, and recombinant proteins**		

Ebselen (sEH dual inhibitor)	Tokyo Chemical Industry Co., Japan	E0946; CAS:60940-34-3
N-acetyl-S-farnesyl-L-Cysteine (sEH dual inhibitor)	Cayman Chemical	62720; CAS: 135304-07-3
AUDA (C-EH inhibitor)	Cayman Chemical	10007927; CAS: 479413-70-2
TPPU (C-EH inhibitor)	Cayman Chemical	11120; CAS: 1222780-33-7
3-amino-4-hydroxy benzoic acid (N-phos inhibitor)	Tokyo Chemical Industry Co., Japan	A0859; CAS: 1571-72-8
Oxaprozin (N-phos inhibitor)	Tokyo Chemical Industry Co., Japan	O0377; CAS:21256-18-8
U73122 (PLC inhibitor)	Sigma-Aldrich	U6756; CAS: 112648-68-7
Manoalide (PLC inhibitor)	Abcam	ab141554; CAS: 75088-80-1
Oleoyl-lysophosphatidic acid (LPAR agonist)	Cayman Chemical	10010093; CAS: 65528-98-5
H2L 5765834 (LPAR antagonist)	Tocris Bioscience	4870; CAS: 420841-84-5
Ki16425 (LPAR antagonist)	MedChemExpress	HY-13285; CAS: 355025-24-0
I-BOP (TXA2R agonist)	Cayman Chemical	19600; CAS: 128719-90-4
Daltroban (TXA2R antagonist)	Cayman Chemical	14061; CAS: 79094-20-5
Picotamide (TXA2R antagonist/TXA2 synthase inhibitor)	Cayman Chemical	21690; CAS: 32828-81-2
Pyrrophenone (cPLA2 inhibitor)	Santa Cruz Biotechnology	sc-296161; CAS: 341973-06-6
Thymoquinone (COX inhibitor)	Tokyo Chemical Industry Co., Japan	T0795; CAS: 490-91-5
Licofelone (COX/LOX inhibitor)	Cayman Chemical	10007692; CAS: 156897-06-2
FR 180204 (ERK inhibitor)	Tokyo Chemical Industry Co., Japan	F1214; CAS: 865362-74-9
TG100-115 (PI3K inhibitor)	FUJIFILM Co., Japan	HY-10111; CAS: 677297-51-7
Y-27632 (ROCK inhibitor)	Cayman Chemical	10005583; CAS: 129830-38-2
Bisindolylmaleimide IV (PKC inhibitor)	TCI Co., Japan	B6068; CAS: 119139-23-0
Torin1 (mTOR inhibitor)	Focus Biomolecules	FCS-10-3013-5; CAS: 1222998-36-8
Lipopolysaccharide	FUJIFILM Co., Japan	125–05201
Macrophage colony stimulating factor	PeproTech	315–02
Interferon-γ	FUJIFILM Co., Japan	094–04701
Interleukin 4	PeproTech	214–14
Interleukin13	PeproTech	210–13

**Critical commercial assays**		

Lipidome analysis (outsourcing)	Human Metabolome Technologies Inc., Japan	No.163-H57
TXB2 ELISA kit	ENZ Enzo life Sciences, Inc.	ADI-900-002
IP-One ELISA kit	CISBIO, Revvity for Life Sciences, Inc.	72IP1PEA
Cell chemotaxis assay chamber	KURABO Industries Ltd.	CH5-24
Latex beads, fluorescent red	Sigma-Aldrich	L3030
Hydrogen peroxide assay kit (Cell-based)	Abcam	ab138874
Mouse TNF-α quantikine ELISA Kit	R&D Systems	MTA00B

**Deposited data**		

Raw and analyzed data	This paper	N/A
Microarray data	The Immunological Genome Project	ImmGen reference set (Gautier et al., 2012, https://doi.org/10.1038/ni.2419; Jakubzick et al., 2013, https://doi.org/10.1016/j.immuni.2013.08.007; Kang et al., 2013, https://doi.org/10.1016/j.it.2013.03.004.)

**Experimental models: Cell lines**		

Raw 264.7 macrophage cell line	RIKEN, Japan	N/A
A20 lymphoma cell line	RIKEN, Japan	N/A

**Experimental models: Organisms/strains**		

Mouse: C57BL/6J	Institute of Medical Science, University of Tokyo	N/A

**Oligonucleotides**		

qPCR Primer: See Table S3	FASMAC Co.,Ltd.	by NCBI primer design tool

**Software and algorithms**		

Laica Application Suite X	Leica Microsystems	https://www.leica-microsystems.com
FV10-ASW Viewer	Olympus	https://www.olympuslifescience.com/en/downloads/detail-iframe/
LightCycler® 480 System	NIPPON Genetics co.,Ltd.	https://www.n-genetics.com/
Fiji ImageJ	US. NIH	https://fiji.sc/
GraphPad Prism 7	GraphPad Software	https://www.graphpad.com/features

### Resource availability

#### Lead contact

Further information and any related requests should be directed to and will be fulfilled by the lead contact, Hiroshi Takeshima (takeshim@pharm.kyoto-u.ac.jp).

#### Materials availability

This paper did not generate new unique reagents.

Antibodies were commercial sources described in the STAR methods key resources table.

### Experimental model and study participant details

#### Chemicals, primers and mice

The reagents and antibodies used in this study are described in the STAR methods key resources table. Synthetic primers used for PCR analysis are listed in Table S3. C57BL/6J mice were purchased from Shimizu Laboratory Supplies (Kyoto, Japan). All experiments in this study were conducted with the approval of the Animal Research Committee according to the regulations on animal experimentation at Kyoto University.

#### Preparation of TGPMs

C57BL/6J male mice (9–11 weeks old) were injected i.p. with 1 mL of 30 mg/mL Brewer thioglycolate (Defco) 3 days before cell preparation. The mice were then injected with phosphate-buffered saline (PBS), and the suspended cells were collected from the peritoneal cavity, recovered by low-speed centrifugation, re-suspended in culture medium (RPMI 1640 supplemented with 10% fetal calf serum and penicillin/streptomycin cocktail) and pre-seeded on plastic dishes for 2 h. After non-adherent cells were removed, adherent TGPMs were scraped off with Cell Spatula (Techno Plastic Products Inc., Switzerland) and then seeded on polylysine-coated glass-bottom dishes (MatTek Co.) for imaging analysis after 20∼26-h culture or on plastic dishes for biochemical assessments after 3-h culture.

#### Isolation and differentiation of BMDMs

From the femoral bones dissected from male C57BL mice (8–10 weeks old), bone marrow cells were flushed out with cold phosphate-buffered saline (1 mL/femur). After passing through mesh filters, isolated bone marrow cells were cultured in DMEM medium supplemented with glutamine, pyruvate, 10% fetal calf serum, 0.5% penicillin/streptomycin cocktail and 50 ng/mL macrophage colony stimulating factor (PeproTech) for 5 days to differentiate into BMDMs. On culture day 5, M1 polarization was achieved by supplementation with 100 ng/mL LPS (Fujifilm Co., Japan) and 20 ng/mL IFN-γ (Fujifilm Co., Japan) for 24 hrs, while M2 polarization was achieved by supplementation with 20 ng/mL interleukin-4 (PeproTech) and 20 ng/mL interleukin-13 (PeproTech) for 24 hrs. M1-and M2-polarizing cells thus generated were subjected to immune-staining and RT-PCR analyses.

### Method details

#### Fura-2 Ca^2+^ imaging

TGPMs were subjected to Fura-2 imaging as described previously.^14^ TGPMs were cultured on the glass-bottom dishes for 18–24 h, and incubated in the culture medium containing 5 μM Fura-2a.m. (Dojindo, Japan) for 30 min. for Ca^2+^ imaging, excitation light at 340 and 380 nm was delivered, and emission light at >510 nm was detected by a CMOS camera (molecular Devices) mounted on a microscope (DMI4000B with an x20 objective, Leica) equipped with a polychrometer (Meta Fluor imaging System, Universal imaging). The bathing solution used was HEPES-buffered saline (107 mM NaCl, 6 mM KCl, 1.2 mM MgSO_4_, 2 mM CaCl_2_, 11.5 mM glucose and 20 mM HEPES, pH 7.4) that was warmed by the cord heater looped around a perfusion tube to maintain the infusion at ∼37°C during the measurements. In typical measurements, 30 cells were randomly selected in each recording dish, and resting levels and triggered shifts in Fura-2 ratio (F_340_/F_380_) were individually determined from their recording traces using the commercial software Suite X (Leica) for statistical analysis

#### RT-PCR analysis

Total RNA was prepared from TGPMs using a commercial kit (Isogen, Nippon Gene Co., Japan) and reverse transcribed using the ReverTra ACE qPCR-RT kit (Toyobo Co., Japan). The resulting cDNA was examined using the primer set listed in Table S3 by real-time PCR (LightCycler 480 II, Roche); cDNA derived from 5 ng of total RNA was used for each reaction. The cycle threshold (*Ct*) was determined from the amplification curve as an index for relative mRNA content in each reaction.

#### Lipidome analysis

TGPMs (10^7^ cells) were treated with or without 10 mM AHBA for 5 min for lipidome analysis using liquid chromatography triple quadrupole mass spectrometry (LC-TQMS) in an outsourcing service provider (Human Metabolome Technologies Inc., Japan; https://en.humanmetabolome.com/). For the Multiphospholipid package analysis, total lipids were prepared by chloroform-methanol extraction from TGPMs, and subjected to diethylaminoethyl-cellulose chromatography to yield phospholipid fractions. The resultant phospholipids were methylated using TMS-diazomethane, and subjected to LC-MS/MS analysis using the Xevo TQ-XS mass spectrometer with an ACQUITY UPLC H-Class (Waters). The phosopholipid species were separated on the X-Bridge C18 column (Waters) and measured using multiple reaction monitoring in positive ion mode. Peak areas of individual species were normalized with those of deuterated internal standards (Avanti polar lipids Inc.), which were added to the samples before lipid extraction. The LC-MS/MS raw data were processed using the analytical MassLynx4.2 software (Waters). For the Eicosa/Docosanoid package analysis, crude lipid extracts were prepared from TGPMs by methanol deproteinization. Fatty acid metabolites were enriched from the extracts by solid phase extraction with Oasis HLB columns (Waters) after adding internal standards (15-HETE-d8, LTB4-d4, PGE2-d4 and AA-d8; Cayman chemicals). Individual metabolites were further separated using a liquid chromatography system (Nexera LC-30AD, Shimadzu Co., Japan) equipped with an XBridge C18 column (Waters) and analyzed on a TQMS (LCMS-8040; Shimadzu). For quantification using LabSolutions software (Shimadzu), calibration curves were prepared for each metabolite and recovery rates were monitored using the deuterated internal standards.

#### Immunoblot analysis

TGPMs were treated with or without AHBA (10 μM for 20 min) and then lysed in RIPA buffer (150 mM NaCl, 1% NP-40, 0.5% deoxycholate, 0.1% sodium dodecyl sulfate and 25 mM Tris-HCl, pH 7.4) supplemented with phosphatase inhibitor cocktail (100 mM NaF, 10 mM Na_3_PO_4_, 1 mM Na_2_VO_3_ and 20 mM β-glycerophosphate). The cell lysates were electrophoresed on SDS–polyacrylamide gels, and the separated proteins were electrophoretically transferred to nylon membranes (polyvinylidene difluoride, Merck Millipore). After being treated with skim milk or blocking reagent (Blocking One-P solution, Nacalai Tesque Co, Japan), the membranes were probed with primary antibodies and then exposed to horseradish peroxidase–labeled secondary antibodies listed in Table S3. Immunoreactivity was visualized using a chemiluminescence reagent (GE Healthcare Life Sciences) and an image analyzer (Amersham Imager 600, GE Healthcare Life Sciences), and was quantitatively analyzed using the ImageJ software (US. NIH).

#### Assessments of TXA2 synthesis and PLC activity

TXA2 production was estimated using a commercial kit (TXB2 ELISA kit, Enzo Life Sciences Inc.) according to the manufacturer’s instructions. TGPMs (2x10^6^ cells) were incubated with or without AHBA (10 μM for 20 min) in fresh culture medium, and the resulting culture supernatant (0.1 mL) was collected to immunochemically measure TXB2 as a surrogate for TXA2 generated during incubation. For pharmacological examinations, several chemical inhibitors were co-treated with AHBA.

Cellular PLC activity was estimated using a commercial kit (IP-one ELISA kit, Cisbio Co.). TGPMs (2 × 10^6^ cells) were incubated with or without AHBA (3 or 10 μM for 20 min) with or without inhibitors in the medium containing 50 mM LiCl to prevent the degradation of inositol monophosphate (IP_1_) into *myo*-inositol. The cells were treated with an adjunctive lysis buffer, and then cellular IP_1_ was immunochemically quantified as a surrogate for IP_3_ generated during incubation.

#### Assessments of cellular functions

AHBA-induced cytotoxicity was examined with a commercial kit (CellTiter 96 Aqueous One Solution Cell Proliferation Assay, Promega). TGPMs (10^4^ cells/well) were cultured with or without AHBA for 24 h in 96-well plates, and then treated with the included reagent for 1 h according the manufacturer’s instructions. The quantity of the formazan product as an index of living cells was colorimetrically measured at an optical density (OD) of 490 nm.

Chemotactic migration was examined using a commercial apparatus (Cell chemotaxis assay chamber, TOSC Japan Ltd.). TGPMs (2×10^6^ cells/well) were seeded into top chambers, which were filled with regular medium, and the chambers were then placed in 24-well plates (bottom chambers) filled with medium containing 100 μM ATP; media in the top and bottom chambers were supplemented with an equal concentration of AHBA to evaluate its effect. After 3-h incubation, cells that migrated to the bottom chamber were recovered by scraping for cell counting.

To examine phagocytic activity, TGPMs (2×10^6^ cells) were cultured in a glass bottom dish for 1 h, and then treated with red-fluorescent latex microbeads (2 μm in diameter, catalog No. L3030, Sigma-Aldrich) at a multiplicity of infection (MOI) of 75:1 for 3 h. After extracellular beads were removed by washing with PBS, TGPMs were formaldehyde-fixed and reacted with Hoechst (Hoechst 33342 solution, Dojindo Co, Japan) and FITC-labeled anti-F4/80 antibody for nuclear and cell-surface staining, respectively. After being washed with PBS, intracellular microbeads in F4/80-positive TGPMs were examined under a fluorescence microscope (BZ-X800, Keyence, Japan). In each assay, 60 cells were randomly selected for quantitative analysis.

H_2_O_2_ production was evaluated with a commercial kit (Hydrogen peroxide assay kit, Aabcam) according to the manufacturer’s instructions. TGPMs were cultured with various combination of chemicals plus the H_2_O_2_-reactive AbGreen indicator for 3 h. Fluorescence images at 490 nm excitation and 525 nm emission were then captured by a fluorescence microscope; the AbGreen indicator emits green fluorescence when reacting with H_2_O_2._ Intracellular fluoresce was digitalized and relatively quantified using ImageJ software.

TNF-α production was examined with a commercial kit (mouse TNF-α Quantikine ELISA kit, R&D Systems). TGPMs (10^6^ cells) were cultured with various combination of chemicals (0.1 μg/mL LPS, 30 μM AHBA, 5 μM H2L5765834 and 1 μM daltroban) for 3 h, and the resulting culture supernatants were subjected to the ERISA assay according to the manufacturer’s instructions.

### Quantification and statistical analysis

All data obtained are presented as the means ± SEM. with *n* values indicating the number of examined mice or cells, and were analyzed using the commercial software Prism 7 (GraphPad Software Inc.). Student’s *t* test was applied for two-group comparison. Multiple comparison tests comparing each group with the control group were evaluated using one-way analysis of variance (ANOVA) with post-hoc Dunnett’s test. Multiple comparison test comparing all two groups were analyzed using the Tukey-Kramer test (for two groups) or the Sidak’s test (for three and more groups). Data distributions were evaluated using the Shapiro-Wilk test. In this study, p < 0.05 was generally considered to be statistically significant.

## Data Availability

• All data for evaluating the contributions in the paper are presented in the paper and/or the supplemental information. • Microarray data based on the ImmGen (Immunological Genome Project) reference set (see key resources table). • Any additional information required to reanalyze the data reported in this paper is available from the lead contact upon request.

## Supplemental information

Document S1. Figures S1–S7 and Table S3

Table S1. Summarized data in multi-phospholipid analysis, related to Figure 3

Table S2. Summarized data in eicosa/docosanoid analysis, related to Figure 3
