# Supplementary material for: Soluble epoxide hydrolase maintains steady-state lipid turnover linked with autocrine signaling in peritoneal macrophages
Source: iScience. 2023 Jul 27;26(8):107465. doi: 10.1016/j.isci.2023.107465 (PMC10433125; doi:10.1016/j.isci.2023.107465)
Supplement: Document S1. Figures S1–S7 and Table S3 [file mmc1.pdf]

iScience, Volume *26*

## Supplemental information

# Soluble epoxide hydrolase maintains steady-state lipid turnover linked with autocrine signaling in peritoneal macrophages

**Feng Liu, Xueying Diao, Haolun Cong, Eriko Suzuki, Keiji Hasumi, and Hiroshi Takeshima**

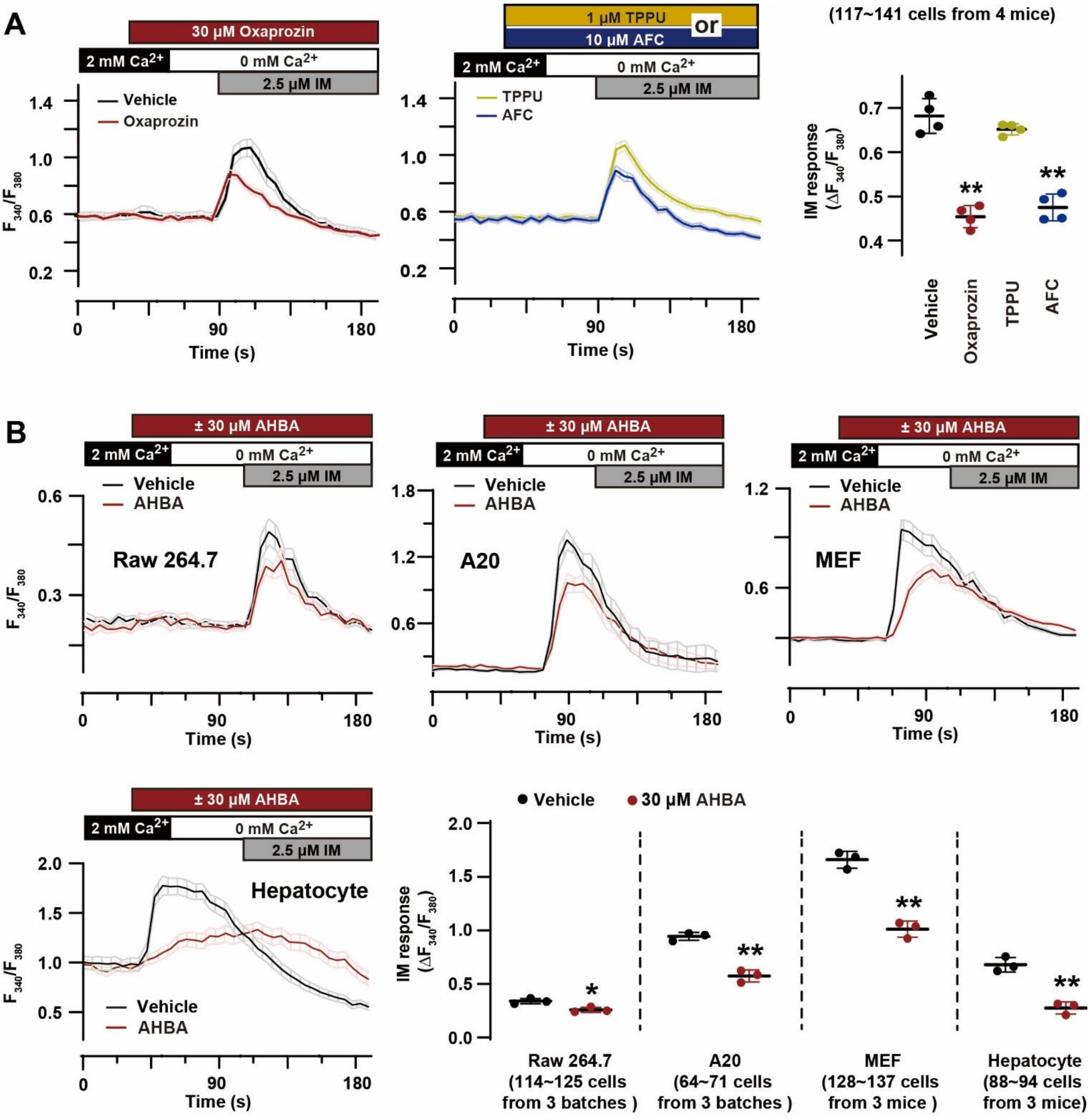


**Figure S1. Reduction of $Ca^{2+}$ stores by N-phos inhibitors, Related to Figure 1**

(**A**) $Ca^{2+}$ store reduction in TGPMs treated with N-phos inhibitors. The Fura-2 imaging traces show averaged time courses with shaded areas indicating standard errors (*n*=10 TGPMs in each group). The inhibitors used are the N-phos inhibitor oxaprozin, the C-EH inhibitor TPPU and the dual inhibitor N-acetyl-S-farnesyl-L-cysteine (AFC). In the dot plot, $Ca^{2+}$ responses evoked by ionomycin (IM) are statistically analyzed, and significant differences from the vehicle-treated group are marked with asterisks (***p* <0.01 in ANOVA followed by Dunnett's test). (**B**) AHBA-induced $Ca^{2+}$ store reduction in several cell types. The imaging traces show averaged time courses with shaded areas indicating standard errors (*n*=10 cells in each group). The cells examined are Raw 264.7 macrophage cell line, A20 lymphoma cell line, primary-cultured mouse embryonic fibroblasts (MEF) and isolated mouse hepatocytes. In the dot plot, IM-induced responses are statistically analyzed in dividual cells, and significant differences from the vehicle-treated groups are marked with asterisks (**p* <0.05 and ***p* <0.01 in *t*-test). The data represent means ± SEM. and the numbers of cells from distinct batches or mice examined are shown in parentheses.

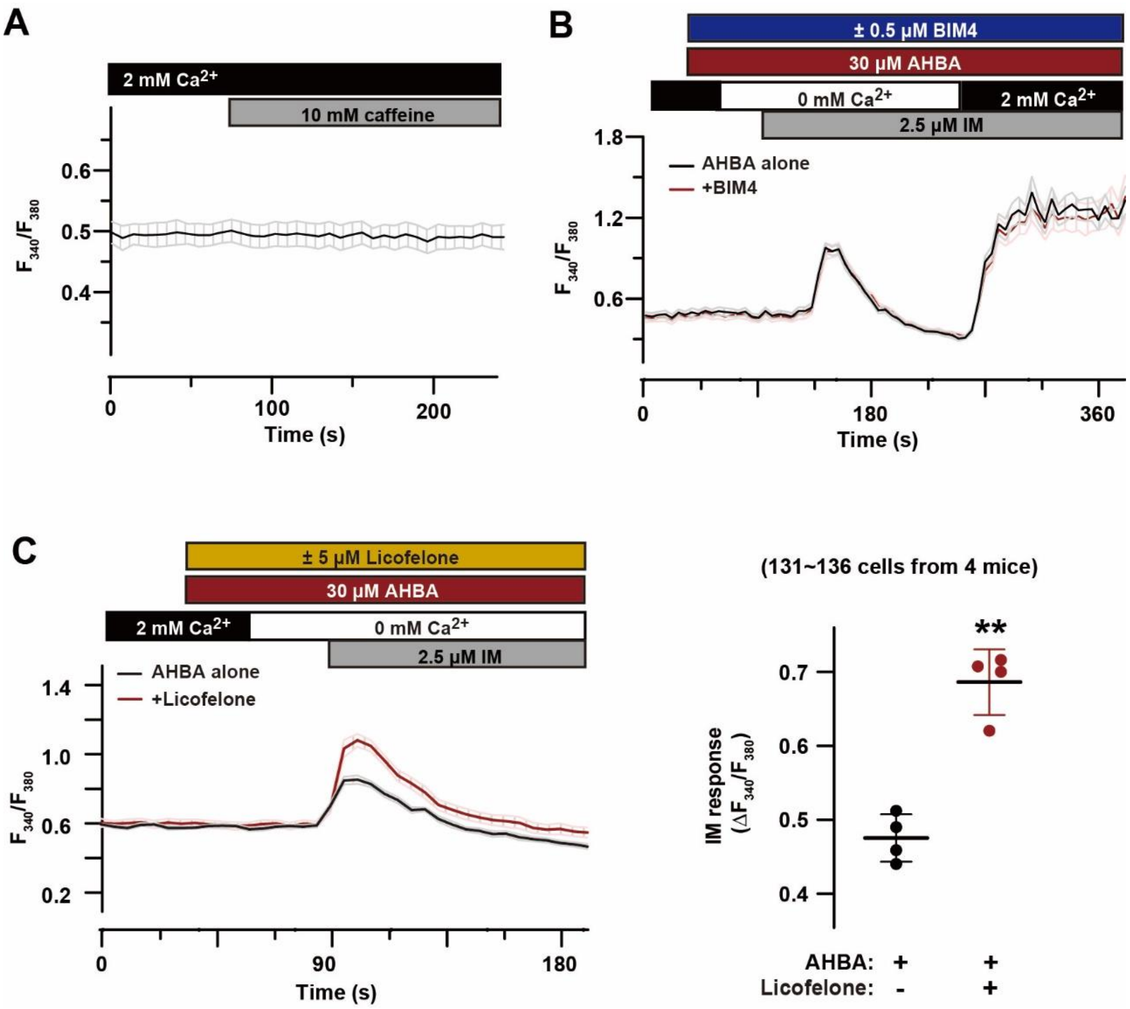


**Figure S2. Some characterization of $Ca^{2+}$ stores in TGPMs, Related to Figures 2 and 6**

(**A**) No caffeine-induced $Ca^{2+}$ release. The Fura-2 imaging traces show averaged time course with shaded area indicating standard error (*n*=10 TGPMs). (**B**) No effect of the PKC inhibitor bisindolylmaleimide IV (BIM4) on AHBA-induced store $Ca^{2+}$ reduction. The imaging traces show averaged time courses with shaded areas indicating standard errors in AHBA-treated TGPMs with or without BIM4 co-treatment (*n*=10 TGPMs in each group). (**C**) Inhibition of AHBA-induced store $Ca^{2+}$ reduction by the COX/LOX inhibitor licofelone. The imaging traces show averaged time courses with shaded areas indicating standard errors in AHBA-treated TGPMs with or without licofelone co-treatment (*n*=10 TGPMs in each group). In the dot plot, $Ca^{2+}$ responses evoked by ionomycin (IM) are statistically analyzed (**$p$ <0.01 in *t*-test). The data represent means ± SEM. and the numbers of cells and mice examined are shown in parentheses.

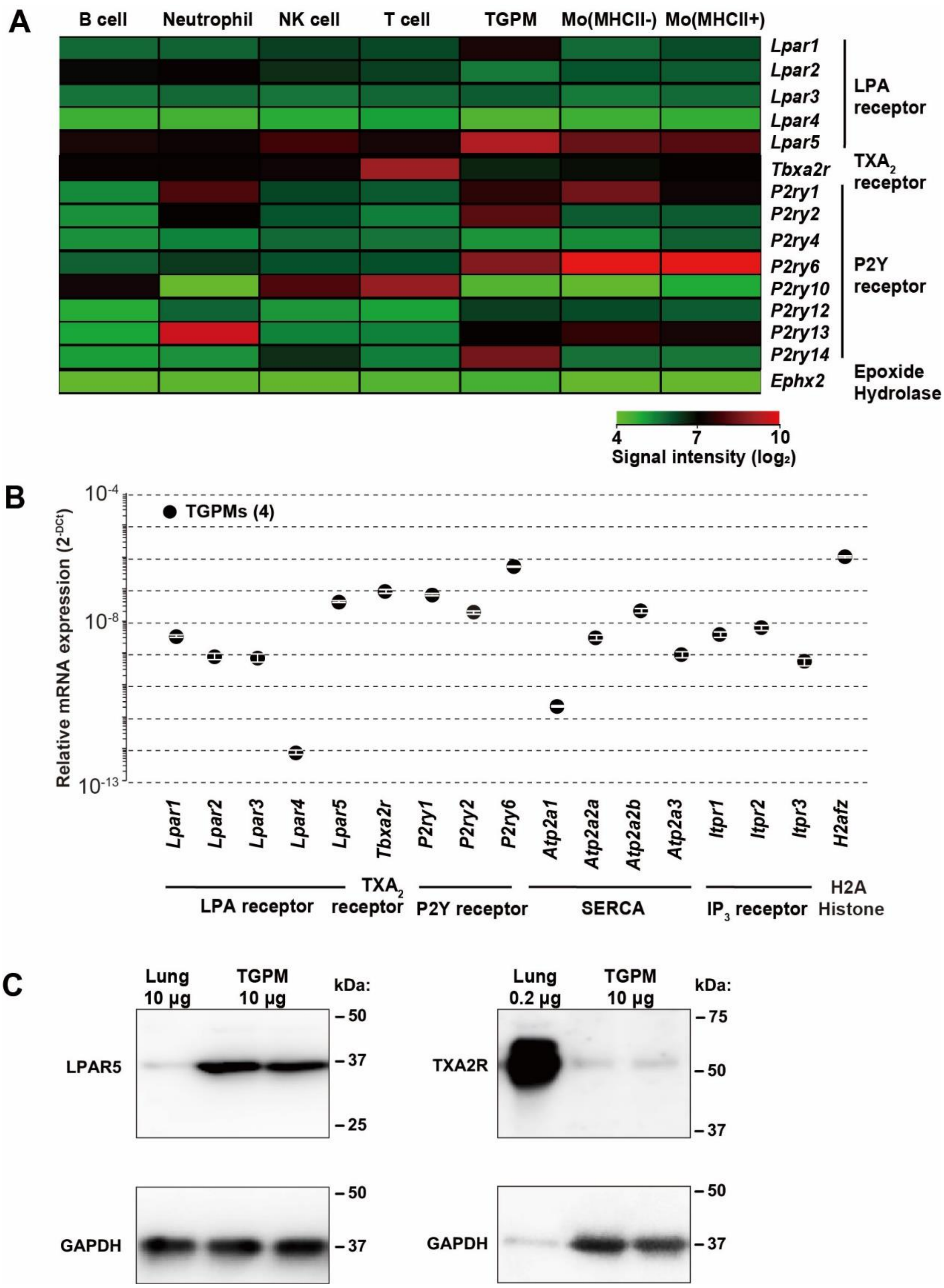


**Figure S3. LPA and TXA2 receptors in TGPMs, Related to Figure 4**

(**A**) Microarray analysis of genes encoding LPA, TXA2 and P2Y receptors in immune cell types. The gene-expression data were taken from the Immgen database (https://www.immgen.org), and are present as the heatmap; green and red denote low and high expression, respectively. Mo, monocyte. MHC, major histocompatibility complex. (**B**) RT-PCR analysis in TGPMs. Total RNAs were reverse-transcribed and the resulting cDNAs were analyzed; the cycle threshold (*Ct*) was determined for each amplification. The data represent the mean ± SEM, and the numbers of mice examined are shown in parentheses. (**C**) Western blot analysis of LPA receptor 5 and TXA2 receptor in TGPMs. Total cell and lung tissue lysates were examined using specific antibodies. Glyceraldehyde-phosphate dehydrogenase (GAPDH) served as a loading control.

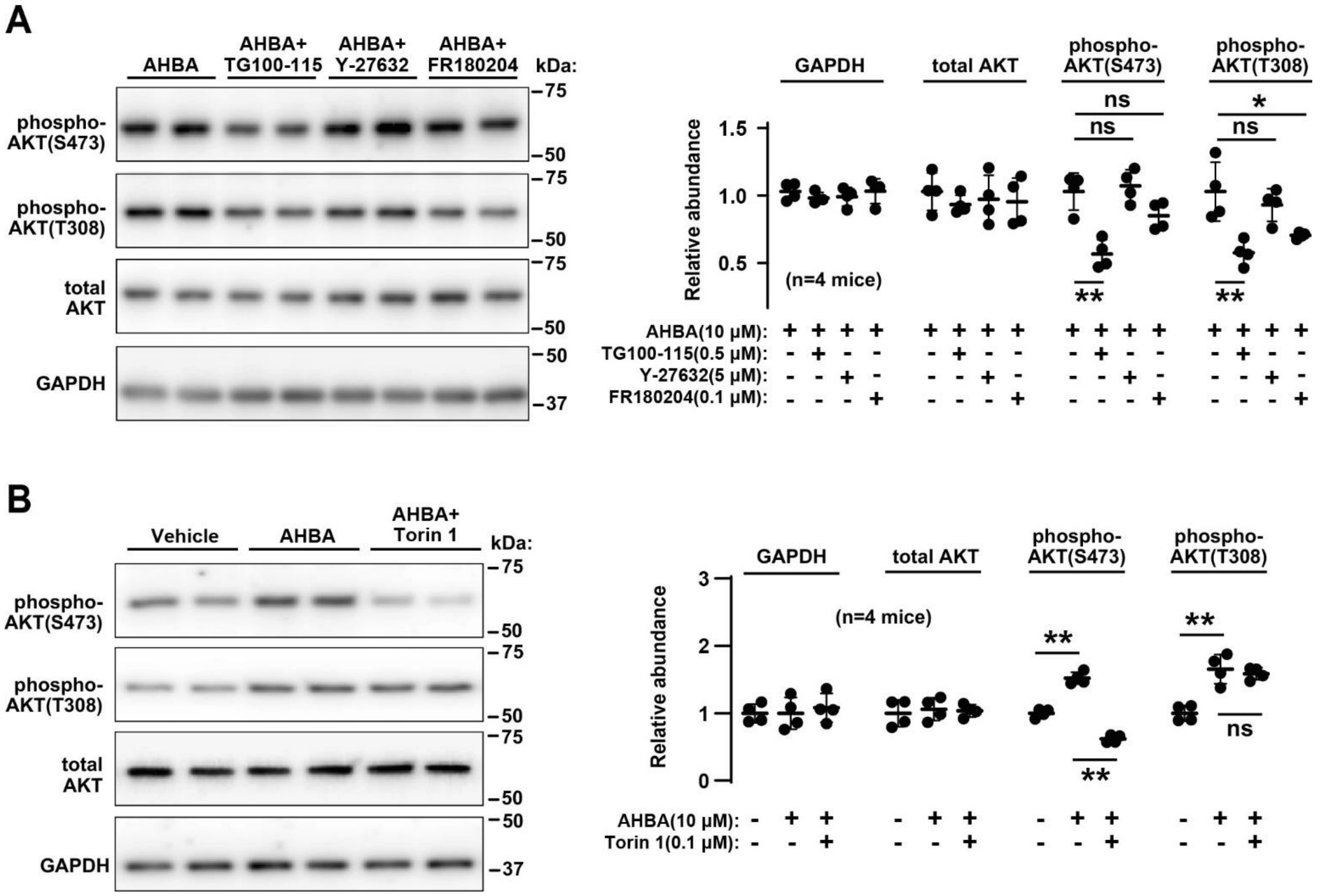


**Figure S4. AHBA-induced AKT phosphorylation, Related to Figure 5**

(**A**) AKT phosphorylation and PI3K. (**B**) AKT phosphorylation and mTOR. The inhibitors used are the PI3K inhibitor TG100-116, the ROCK inhibitor Y-27632, the ERK inhibitor FR 180204 and the mTOR inhibitor Torin 1. Total cell lysates were prepared from TGPMs treated with the combination of inhibitors for 20 min. and subjected to immune-blot analysis. The resulting immune-signals were captured as digital images and quantitively analyzed in the dot plots. The data represent means ± SEM., and the mice examined are shown in parentheses. Statistical differences are examined between the groups indicated by bars (*$p$ <0.05, **$p$ <0.01 and ns: not significant in ANOVA and Sidak's test).

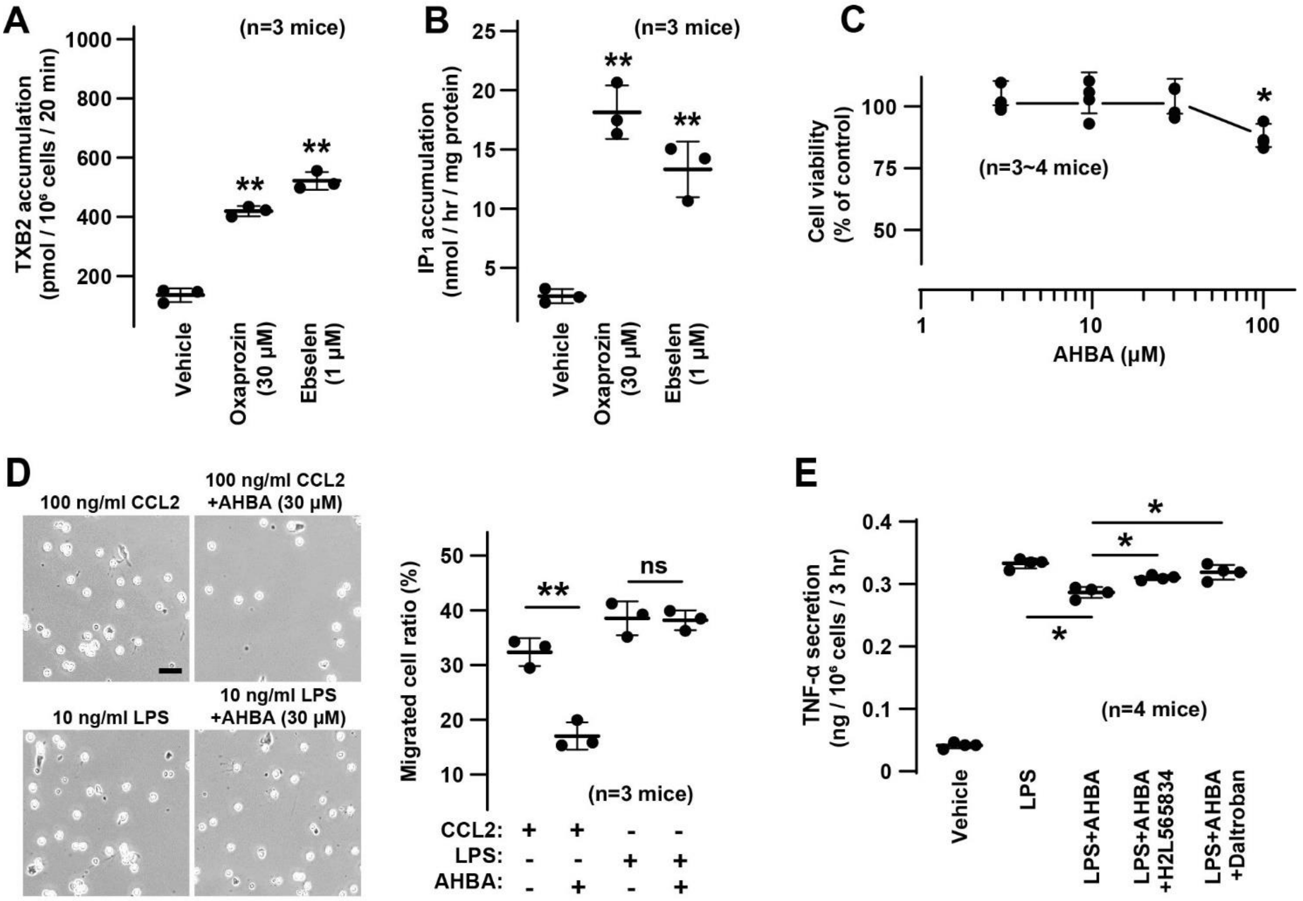


**Figure S5. N-phos inhibiton-induced effects on cellular functions in TGPMs, Related to Figures 7 and 8**

(**A**) N-phos inhibiton-induced TXB2 accumulation. The inhibitors used are the N-phos inhibitor oxaprozin and the sEH dual inhibitor ebselen. TGPMs were treated with or without the inhibitors for 20 min, and TXB2 contents in culture supernatants were immunochemically quantified. (**B**) N-phos inhibiton-induced $IP_1$ accumulation. TGPMs were treated with or without the inhibitors for 20 min, and intracellular $IP_1$ contents were immunochemically quantified. (**C**) No obvious toxic effects of AHBA. TGPMs were treated with AHBA at several doses for 24 hr and subjected to the MTT assay determining cell viability. The results obtained (OD 490 nm) were normalized to the corresponding control value from vehicle-treated TGPMs, which is set at 100% in the plot graph. (**D**) Effects of AHBA on chemotaxis to LPS and chemokine C-C motif ligand 2 (CCL2). TGPMs that migrated to the bottom chamber were observed (image panels, scale bar = 25 μm), and the migration capacity was statistically analyzed (right graph). (**E**) AHBA-induced attenuation of TNF-α secretion in LPS-treated TGPMs. TGPMs were treated with the indicated combinations of the inhibitors and together with or without 0.1 μg/ml LPS for 3 hr, and the resulting culture supernatants were examined for TNF-α quantification. The inhibitors used are AHBA (30 μM), the LPA receptor blocker H2L5765834 (5 μM) and the TXA2 receptor blocker daltroban (1 μM). The numbers of mice examined are shown in parentheses, and the data represent means ± SEM. Statistical differences between groups indicated by bars are marked with asterisks (*p <0.05, **p <0.01 and ns, not significant in ANOVA followed by Dunnett's test (**A**, **B**, **C**, **E**) or Tukey-Kramer test (**D**)).

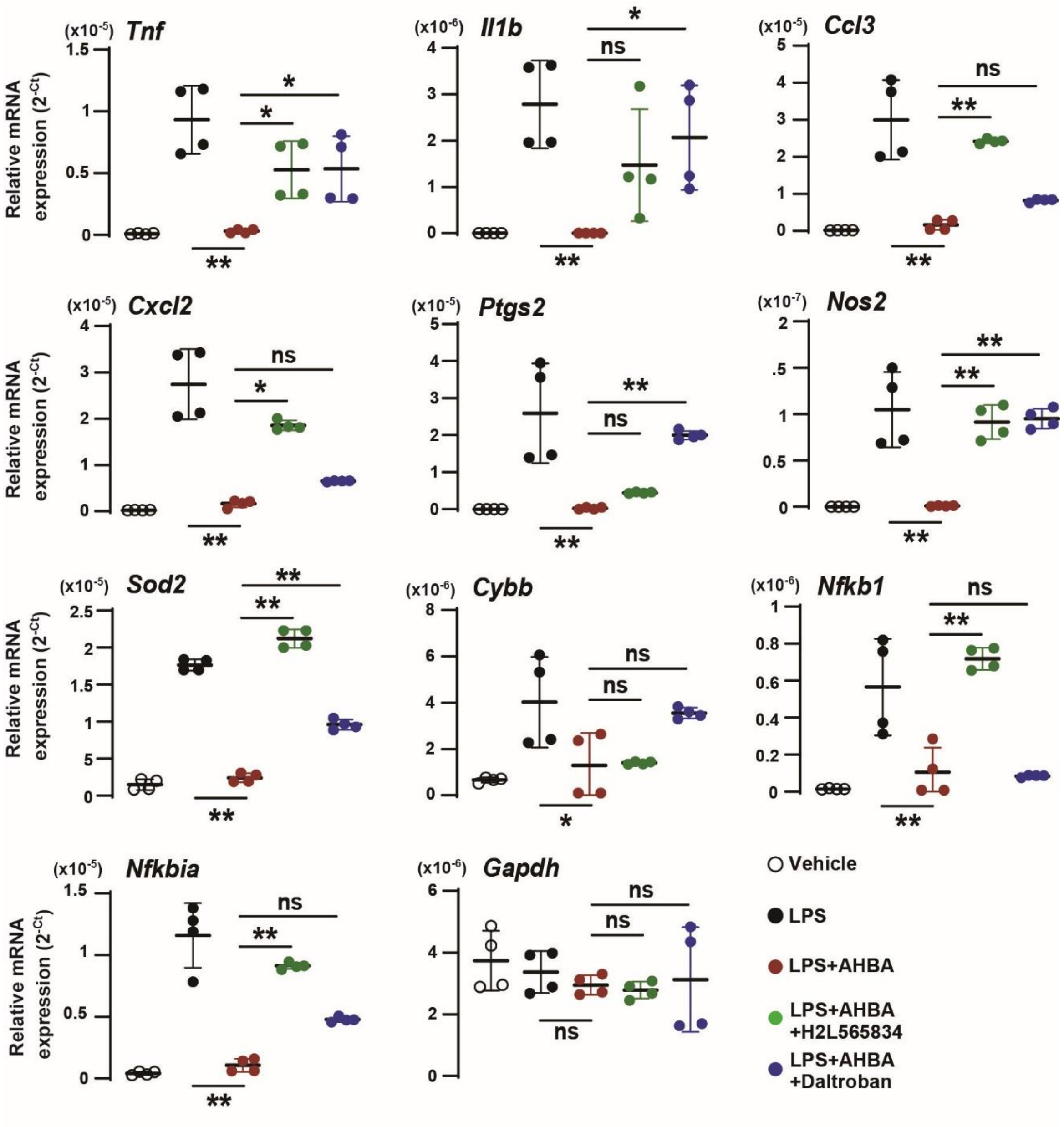


**Figure S6. AHBA-induced attenuation of LPS-facilitated gene expression, Related to Figure 8**

TGPMs were treated with or without LPS (1 μg/ml) for 4 hr and subjected to total RNA preparation for RT-PCR analysis. To examine the effects of inhibitors, TGPMs were exposed to combination of the indicated inhibitors for 20 min, and then treated with LPS. The inhibitors used are AHBA (30 μM), the LPA receptor antagonist H2L565834 (5 μM) and the TXA2 receptor antagonist daltroban (1 μM). The relative gene expression was analyzed using the conventional 2−ΔΔCt method. The genes analyzed are *Tnf* (tumor necrosis factor-α), *Il1b* (interleukin-1β), *Ccl3* (C-C motif chemokine ligand 3), *Cxcl2* (C-X-C motif chemokine ligand 2), *Ptgs2* (cyclooxygenase 2), *Nos2* (nitric oxide synthase 2), *Sod2* (superoxide dismutase 2), *Cybb* (NADPH Oxidase 2), *Nfkb1* (NF-κB subunit 1), *Nfkbia* (NF-κB Inhibitor α) and the house-keeping gene *Gapdh* (glyceraldehyde-3-phosphate dehydrogenase). The numbers of mice examined are shown in parentheses, and the data represent means ± SEM. Statistical differences between groups indicated by bars are marked by asterisks (**p* <0.05, ***p* <0.01 and ns, not significant in ANOVA and Dunnett's test).

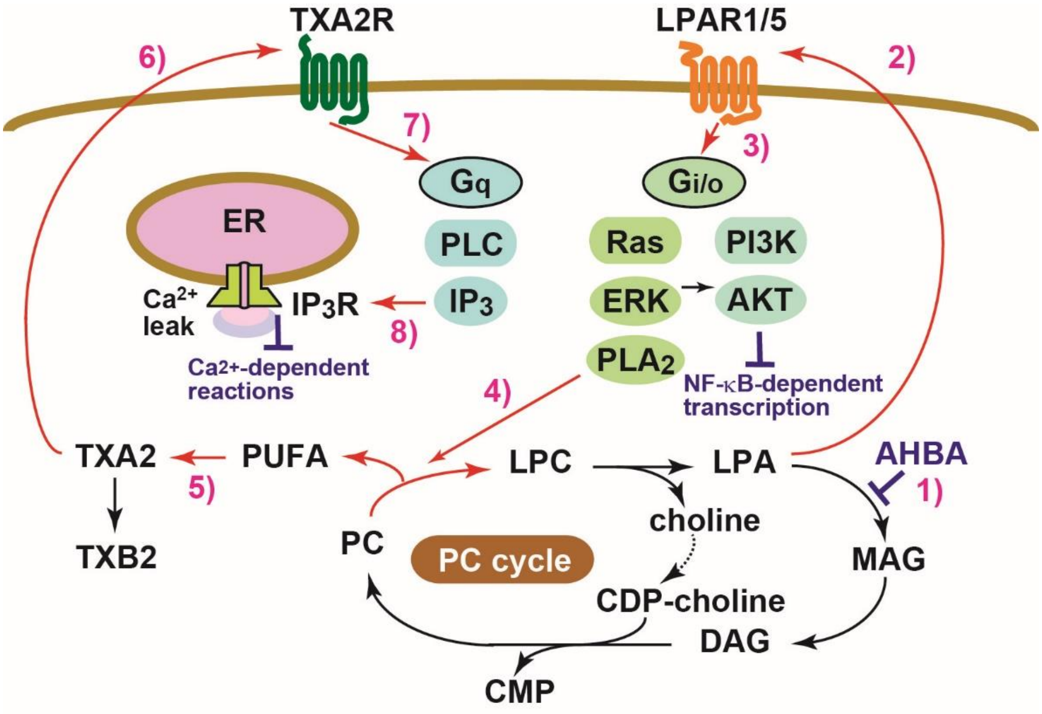


**Figure S7. Proposed scheme for AHBA-induced signaling in TGPMs, Related to Figures 1~9**

Hypothetical mechanism underlying AHBA-induced reduction in $Ca^{2+}$ stores is schematically illustrated. For the details, see the discussion section.

## Table S3. Primers used for RT-PCR analysis, Related to Figures 4 and 9

| | | | |
|---|---|---|---|
| *Atp2a1* | For: CAAAACAGGGACCCTCACCA<br>Rev: GCCAGTGATGGAGAACTCGT | *Atp2a2a* | For: TCTACGTGGAACCTTTGCCG<br>Rev: TGCAAATGGTTTAGGAAGCGG |
| *Atp2a2a* | For: TCTACGTGGAACCTTTGCCG<br>Rev: GCTGCACACACTCTTTACCG | *Atp2a3* | For: CCTCGGTCATCTGCTCTGAC<br>Rev: CGTGGTACCCGAAATGGTGA |
| *Itpr1* | For: GGCAGCATCCAGAGACTACC<br>Rev: GGGGAAGAGCAGTTCTGGTC | *Itpr2* | For: ACAACTGTACCTGGCCCAAC<br>Rev: CTGCACCGTACTTGAGTGGT |
| *Itpr3* | For: AACTACAAGACGGCCACCAG<br>Rev: AGAGCTCAGCCTGTACCAGA | *Lpar1* | For: GCTTGGTGCCTTTATTGTCT<br>Rev: GGTAGGAGTAGATGATGGGG |
| *Lpar2* | For: AGTGTGCTGGTATTGCTGAC<br>Rev: TTTGATGGAGAGCCTGGCAG | *Lpar3* | For: ACTTTCCCTTCTACTACCTG<br>Rev: GTCTTTCCACAGCAATAACC |
| *Lpar4* | For: CCTCAGTGGTGGTATTTCAG<br>Rev: CACAGAAGAACAAGAAACAT | *Lpar5* | For: AACACGACTTCTACCAACAG<br>Rev: AAGACCCAGAGAGCCAGAGC |
| *Lpar6* | For: TACTTTGCCATTTCGGATTT<br>Rev: GCACTTCCTCCCATCACTGT | *Tbxa2r* | For: CCGCTCCTCCTTCCTCGCTCTG<br>Rev: CCACACCAGCCCGACAGTAGCC |
| *Nos2* | For: CTCATTGGGCCTGGTACGG<br>Rev: ACCTGGAACAGCACTCTCTTG | *Cybb* | For: GACTGCGGAGAGTTTGGAAGA<br>Rev: CTGAGATCGCCAAAACCGAAC |
| *Sod2* | For: AAGGGTGGTGGAGAACCCAA<br>Rev: GCAGCAATCTGTAAGCGACC | *Ptgs2* | For: ATCCCCTTCCTGCGAAGTTTA<br>Rev: AGTTGGGCAGTCATCTGCTAC |
| *Tnf* | For: GAACTGGCAGAAGAGGCACT<br>Rev: GAGGCCATTTGGGAACTTCT | *Il1b* | For: GGGCCTCAAAGGAAAGAATC<br>Rev: TACCAGTTGGGGAACTCTGC |
| *Ccl3* | For: AAGGATACAAGCAGCAGCGAGTA<br>Rev: TGCAGAGTGTCATGGTACAGAGAA | *Cxcl2* | For: ACCCCACTGCGCCCAGACAGAA<br>Rev: AGCAGCCCAGGCTCCTCCTTTC |
| *Nfkb1* | For: TGGGAAGGATTTGGGGACTTT<br>Rev: CGAAGCTGAACAAACACGGAA | *Nfkbia* | For: GACCTGGCCTTCCTCAACTTC<br>Rev: CTGCGTCAAGACTGCTACACT |
| *P2ry1* | For: TTGGCTCTGGCTGACTTTTT<br>Rev: CTGTACCTGTGTGCGCTGAT | *P2ry2* | For: CGTGCTCTACTTCGTCACCA<br>Rev: TGGCCATAAGCACGTAACAG |
| *P2ry6* | For: CATTAGCTTCCAGCGCTACC<br>Rev: GCTCAGGTCGTAGCACACAG | *Cd86* | For: ACAAGAAGCCGAATCAGCCTA<br>Rev: CTGAAGCAATTTGGGGTTCAAGT |
| *Cd206* | For: CTGCAAGGAAGGTTGGCATTT<br>Rev: AGGTTTCCTTTCAGTCCTTTGC | *Ym1* | For: ACTTTGATGGCCTCAACCTG<br>Rev: AATGATTCCTGCTCCTGTGG |
| *Arg1* | For: ATGGGCAACCTGTGTCCTTT<br>Rev: GTCTACGTCTCGCAAGCCAA | *Gapdh* | For: TGTGTCCGTCGTGGATCTGA<br>Rev: TTGCTGTTGAAGTCGCAGGAG |
| *H2afz* | For: TAGGACAACCAGCCACGGA<br>Rev: TGACGAGGGGTGATACGCTT | | |
